# The balance between NANOG and SOX17 mediated by TET proteins regulates specification of human primordial germ cell fate

**DOI:** 10.1186/s13578-022-00917-0

**Published:** 2022-11-04

**Authors:** Zili Li, Fang Fang, Yuting Long, Qian Zhao, Xiaotong Wang, Zhen Ye, Tianqing Meng, Xiuli Gu, Wenpei Xiang, Chengliang Xiong, Honggang Li

**Affiliations:** 1 Institute of Reproductive Health, Tongji Medical College, Huazhong University of Science and Technology, 13 Hangkong Road, Wuhan, 430030 China; 2 Department of Obstetrics and Gynecology, Union Hospital, Tongji Medical College, Huazhong University of Science and Technology, Wuhan, 430022 China; 3 Wuhan Tongji Reproductive Medicine Hospital, 128 Sanyang Road, Wuhan, 430013 China

## Abstract

**Background:**

Human primordial germ cells (hPGCs) initiate from the early post-implantation embryo at week 2–3 and undergo epigenetic reprogramming during development. However, the regulatory mechanism of DNA methylation during hPGC specification is still largely unknown due to the difficulties in analyzing early human embryos. Using an in vitro model of hPGC induction, we found a novel function of TET proteins and NANOG in the hPGC specification which was different from that discovered in mice.

**Methods:**

Using the CRISPR–Cas9 system, we generated a set of *TET1*, *TET2* and *TET3* knockout H1 human embryonic stem cell (hESC) lines bearing a BLIMP1-2A-mKate2 reporter. We determined the global mRNA transcription and DNA methylation profiles of pluripotent cells and induced hPGC-like cells (hPGCLCs) by RNA-seq and whole-genome bisulfite sequencing (WGBS) to reveal the involved signaling pathways after TET proteins knockout. ChIP-qPCR was performed to verify the binding of TET and NANOG proteins in the *SOX17* promoter. Real-time quantitative PCR, western blot and immunofluorescence were performed to measure gene expression at mRNA and protein levels. The efficiency of hPGC induction was evaluated by FACS.

**Results:**

In humans, *TET1*, *TET2* and *TET3* triple-knockout (TKO) human embryonic stem cells (hESCs) impaired the NODAL signaling pathway and impeded hPGC specification in vitro, while the hyperactivated NODAL signaling pathway led to gastrulation failure when Tet proteins were inactivated in mouse. Specifically, TET proteins stimulated *SOX17* through the NODAL signaling pathway and directly regulates NANOG expression at the onset of hPGCLCs induction. Notably, NANOG could bind to *SOX17* promoter to regulate its expression in hPGCLCs specification. Furthermore, in TKO hESCs, DNMT3B-mediated hypermethylation of the NODAL signaling-related genes and *NANOG/SOX17* promoters repressed their activation and inhibited hPGCLC induction. Knockout of *DNMT3B* in TKO hESCs partially restored NODAL signaling and *NANOG*/*SOX17* expression, and rescued hPGCLC induction.

**Conclusion:**

Our results show that TETs-mediated oxidation of 5-methylcytosine modulates the NODAL signaling pathway and its downstream genes, *NANOG* and *SOX17*, by promoting demethylation in opposition to DNMT3B-mediated methylation, suggesting that the epigenetic balance of DNA methylation and demethylation in key genes plays a fundamental role in early hPGC specification.

**Supplementary Information:**

The online version contains supplementary material available at 10.1186/s13578-022-00917-0.

## Introduction

In mammalians, the fetal germ cells undergo two waves of genome-wide reprogramming of DNA methylation to re-establish an epigenetic ground state during the early embryo development process [1, 2]. This process includes cytosine methylation by DNA methyltransferases (DNMTs) and oxidation of 5-methylcytosine (5mC) by the Ten-eleven translocation (TET) family of dioxygenases, which leads to the demethylation of DNA [3, 4]. Recent studies have investigated gene expression landscapes and genome-wide DNA demethylation dynamics in human germ cells [5–9].

Primordial germ cells (PGCs) are the precursors of oocytes and sperm. The epigenetic reprogramming of human PGCs (hPGCs) is similar to that of mouse PGCs and pig PGCs [10–12]. As for DNA methylation, hPGCs exhibit substantial demethylation as early as week 5 of development around their colonization of embryonic gonads. At approximately 10–11 weeks after gestation, the global DNA methylation levels of hPGCs reach the lowest point, with only 6%-7% (median level) residual methylation left in the genome [13]. As a consequence, hPGCs exhibit much lower genome-wide 5mC levels than inner cell mass (ICM) cells of the blastocysts [14]. Meanwhile, there are still some regions that evade genome-wide DNA demethylation. The major families of repetitive elements such as long-interspersed nuclear elements (LINEs), short-interspersed nuclear elements (SINEs) and α satellites still retain abundant residual DNA methylation (~ 12%–37%), and these DNA demethylation ‘‘escapees’’ may contribute to the transgenerational epigenetic inheritance and the Hominidae-specific Transposable Elements (TEs) LTR5Hs may serve as TEENhancers (TE Embedded eNhancers) to facilitate PGC specification [6, 15, 16].

Notably, hPGCs appear shortly after blastocyst implantation at around week 2 of development, a stage untouchable to analyze due to both technical difficulties and ethical restrictions [17]. But this process can be modeled with human pluripotent stem cells (hPSCs) by differentiating them into incipient mesoderm-like cells (iMeLCs), which could be robustly induced into hPGC-like cells (hPGCLCs) *in vitr*o [18–20]. Based on this experimental system, Saito et al*.* uncovered a unique transcriptional architecture for human germ cell specification. *EOMES* (*T* in mouse), a downstream TF (transcription factor) of the WNT signaling, activates *SOX17*, which upregulates *BLIMP1* and other hPGCLCs programs. Independent from *SOX17*, *TFAP2C* is initially activated through the BMP signaling and works together with *SOX17* to establish the gene expression program of hPGCLCs in an interdependent fashion, which is different from mice results [21–23]. In addition to in vitro studies, recent high-throughput sequencing data on preimplantation embryos have also provided some insights on hPGC specification [24, 25], but our knowledge of human germline development is still substantially incomplete. Especially, the mechanism of epigenetic regulation at the initiation of hPGC specification remains unclear.

To explore the role of TET-mediated DNA demethylation in hPGC specification, we inactivated all three TET genes in hESC lines (TKO hESCs) and found that in the absence of TET proteins, the de novo methyltransferase DNMT3B caused aberrant hypermethylation of *NANOG* and *SOX17* promoters, which resulted in impaired gene activation and defects in hPGC induction in vitro. Additionally, RNA sequencing (RNA-seq) and whole-genome bisulfite sequencing (WGBS) revealed that LEFTY-NODAL signaling only governed the expression of *SOX17* rather than *NANOG* during hPGC specification. And the balance between SOX17 and NANOG meditated by the TET/DNMT3B proteins is critical for hPGC differentiation, which presents an important advance in epigenetic regulation of human germline development.

## Results

### TET proteins are critical for hPGCLC differentiation

To explore the role of TET-mediated demethylation in hPGC specification, we used the CRISPR-Cas9 system to generate a set of *TET1*, *TET2* and *TET3* knockout H1 hESC lines bearing a BLIMP1-2A-mKate2 reporter, which we established before to optimize hPGCLCs induction methods (Additional file 1: Fig. S1A). The BLIMP1-2A-mKate reporter activates the expression of mKate2 upon *BLIMP1* expression [26]. The knockout cells were analyzed for targeted mutations of the relevant loci by DNA sequencing, and the efficiency of knockout were varied with different sgRNAs (Additional file 1: Fig. S1B–D). TET1 single KO cells and TKO cells exhibit separate mutation sites at the *TET1* gene (Additional file 1: Fig. S1C), which reduces the chance of off-target effects causing the following phenotype.

**Fig. 1:**
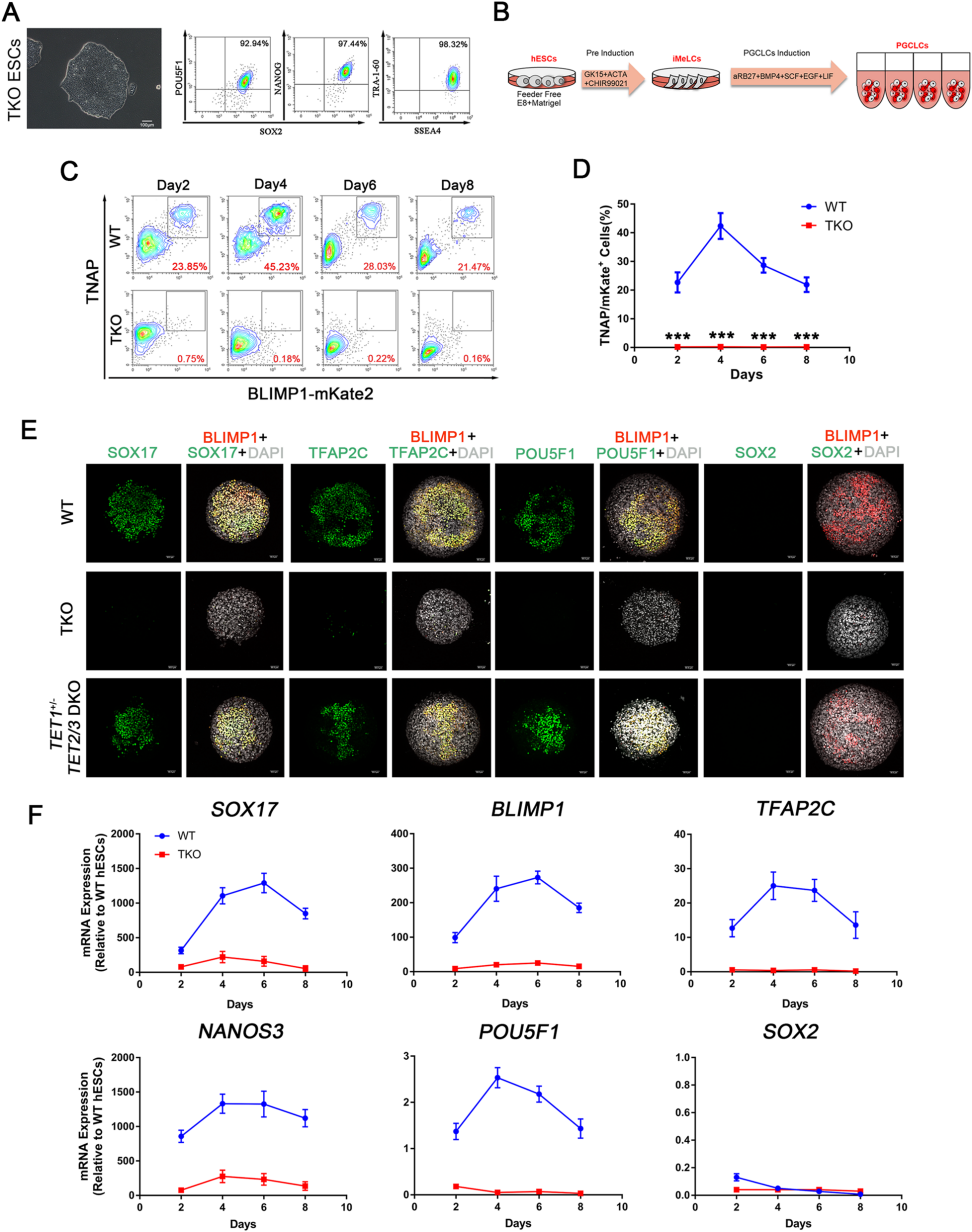
*TET* TKO hESCs Exhibit hPGC Differentiation Defects. **A** Left, A phase-contrast image of TKO hESCs. Scale bar = 100 μm. Right, FACS analysis for POU5F1, SOX2, NANOG, TRA-1-60 and SSEA-4 expression in TKO hESCs; **B** Scheme of hPGC differentiation through iMeLCs in vitro; **C** FACS analysis of WT and TKO hESCs on hPGCLCs induction for 8 days. Boxed areas indicate TNAP/BLIMP1 (+) cells with their percentages. **D** Quantification of TNAP/BLIMP1 (+) cells at day 2, 4, 6 and 8 of hPGC induction; n = 3 independent experiments. Data are presented as means ± s.d. Statistical analysis was performed by Student’s *t-*test (two-sided), ***represent compared to WT group *p* < 0.001; **E** Immunofluorescence of SOX17, TFAP2C, POU5F1, BLIMP1 and SOX2 at the day4 embryoids for WT and TKO cells. Scale bar = 50 μm; **F** RT-qPCR analysis for *SOX17, BLIMP1, TFAP2C, NANOS3, POU5F1* and *SOX2* during hPGC differentiation in day 2 ~ 8 embryoids; n = 3 independent experiments. Data are presented as means ± s.d

Our results showed that the 5-hydroxymethylcytosine (5hmC) levels were dramatically decreased when *TET1* was inactivated, and no 5hmC signal was detected by dot blot in TKO hESCs. But all the cell lines exhibited no difference in 5mC levels (Additional file 2: Fig. S2A, B). Meanwhile, TKO hESCs still maintained normal morphology, and expressed pluripotency markers, such as NANOG, SOX2 and POU5F1 (Fig. 1A). And TKO hESCs showed no difference in proliferation ability compared with WT hESCs (Additional file 2: Fig. S2C). We applied our optimized method to generate hPGCLCs (Fig. 1B), however, TKO hESCs displayed a complete inability to form TNAP (tissue-nonspecific alkaline phosphatase, a PGC and pluripotency marker in humans and mice)/BLIMP1 double-positive hPGCLCs from day 2 to day 8 of induction as determined by FACS (Fig. 1C, D). And activation of key PGC genes like *SOX17*, *TFAP2C*, *NANOS3*, *BLIMP1* and *POU5F1* (also known as *OCT4*) was repressed upon hPGCs induction from TKO hESCs, suggesting that the TET proteins are necessary for the initiation of hPGC specification (Fig. 1F). But the expression of *SOX2*, which is upregulated in mPGCs, was downregulated in both TKO and WT hESCs upon hPGCLC induction (Fig. 1F). During the hPGCLC induction process, the percentage of BLIMP1-mKate2 positive cells increased progressively until day 4, resulting in 37% ~ 50% of TNAP/BLIMP1 double-positive putative hPGCLCs. Similar to other studies, hPGCLCs did not proliferate significantly after day 4 of induction (Fig. 1D). Immunofluorescence confirmed that BLIMP1-mKate2 expression coincided with POU5F1, SOX17 and TFAP2C in day 4 embryoids (Fig. 1E).

Interestingly, the efficiency of hPGCLC induction from TET1-inactivated cell lines was significantly lower than that of WT hESCs, but *TET2* and/or *TET3* knockout did not affect hPGCLCs formation in vitro (Additional file 3: Fig. S3A–D, Additional file 4: Fig. S4). Meanwhile, there is no difference in morphology and key gene expression between TKO and WT hESCs after iMeLCs induction (Additional file 5: Fig. S5A, B). Considering TKO hESCs completely abolished hPGCLC formation, we infer that hPGCLC induction from hESCs is dominantly regulated by TET1-mediated DNA demethylation, and TET proteins act complementarily to orchestrate the epigenetic regulation in the hPGC differentiation process.

### Inactivation of TET proteins impairs LEFTY-NODAL signaling pathway during hPGCLC specification

**Fig. 2:**
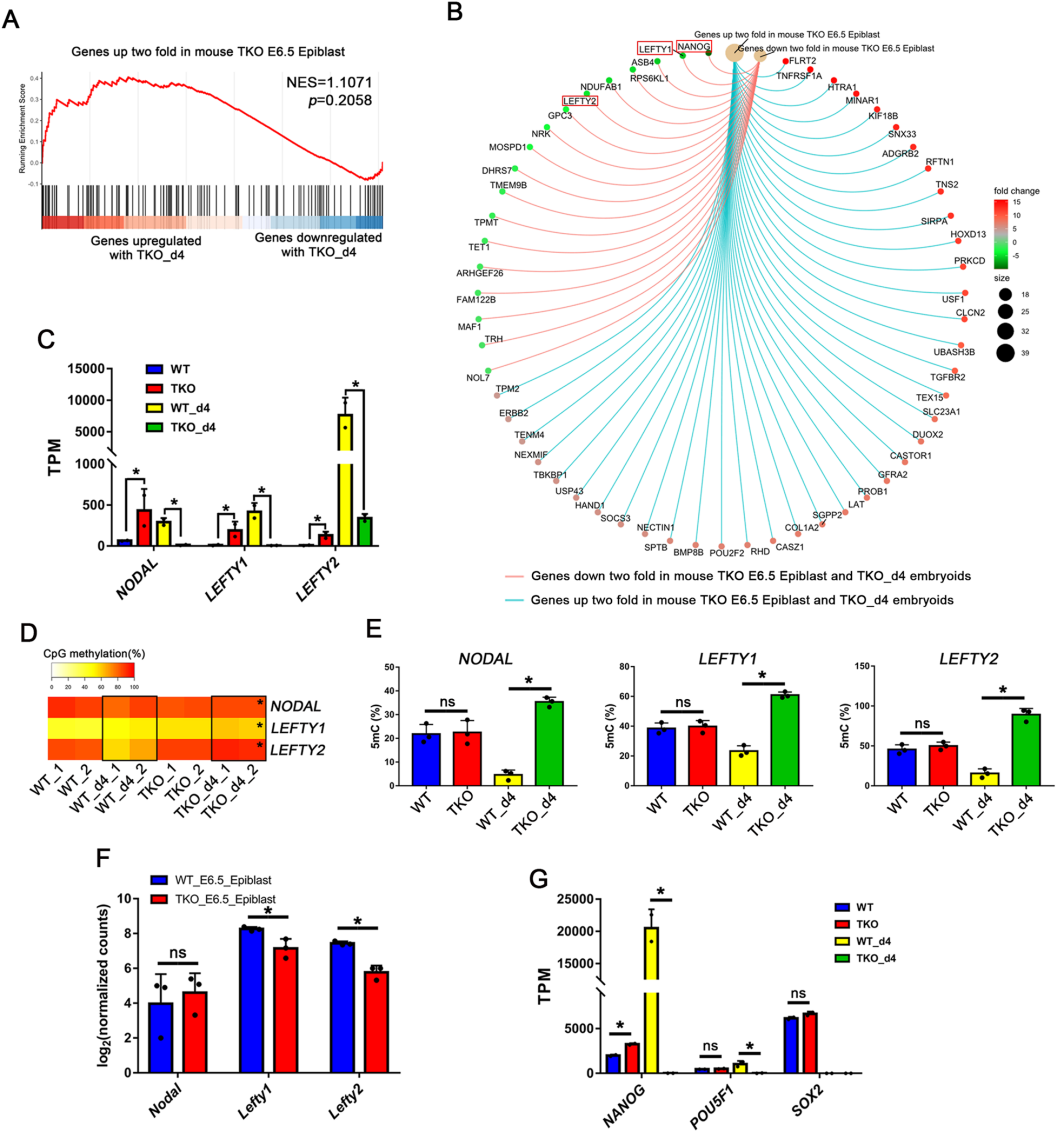
Impaired LEFTY-NODAL Signaling Pathway in TKO hESCs. **A** Gene set enrichment analysis (GSEA) highlights the similarities between TKO day4 embryoids and mouse *Tet*-TKO E6.5 epiblasts. Genes upregulated in *Tet*-TKO E6.5 epiblasts are highly biased to be upregulated in TKO day4 embryoids; **B** Upregulated or downregulated genes between TKO day4 embryoids and mouse *Tet*-TKO E6.5 epiblasts by GSEA. *LEFTY1, LEFTY2* and *NANOG* were highlighted by red box; **C** RNA-seq TPM of *NODAL*, *LEFTY1* and *LEFTY2* in each cell line; **D** Heat map showing DNA demethylation dynamics of *NODAL*, *LEFTY1*, *LEFTY2* promoters in each sample, black boxes indicate the differential methylation level, * represent compared to WT_d4 group *p* < 0.05; **E** Analysis of the percentage of 5mC at the *NODAL*, *LEFTY1*, *LEFTY2* promoters by Epimark in hESCs and day 4 embryoids upon hPGCs induction; n = 3 independent experiments. Data are presented as means ± s.d. Statistical analysis was performed by Student’s *t-*test (two-sided): * represent compared to WT_d4 group *p* < 0.05. **F** RNA-seq counts of *Nodal*, *Lefty1*, *Lefty2* in mice WT E6.25 epiblast and TKO E6.25 epiblast; **G** RNA-seq TPM of *NANOG*, *POU5F1*, *SOX2* in hESC, WT day4 hPGCLCs and TKO day4 embryoids

Owing to difficulties in studying human embryos without TET proteins, we compared the RNA-seq data of mouse E6.5 *Tet*-null epiblasts (where and when mice PGCs are first specified) and that of TKO day4 embryoids [27]. Gene set enrichment analysis (GSEA) highlights the similarities between mouse E6.5 *Tet*-null epiblasts and TKO day4 embryoids (Fig. 2A, B). Genes that were up- or down-regulated in mouse E6.5 *Tet*-null epiblasts were highly biased to be up- or down-regulated in TKO day4 embryoids, respectively. Lefty1 and Lefty2 are members of the TGF-β superfamily and antagonize the Nodal signaling that is essential for primitive streak and mesoderm development in mice [28, 29]. Interestingly, they were among the significantly downregulated genes in TKO day4 embryoids (Fig. 2B), but NODAL was also decreased during hPGCLC differentiation (Fig. 2C, Additional file 6: Fig. S6I), and the promoters of *NODAL* and *LEFTY1/2* were hypermethylated (Fig. 2D, E). This is different from *Tet*-null mouse epiblasts in which increased Nodal signaling was observed, probably due to diminished expression of *Lefty1* and *Lefty2* genes (Fig. 2F). But in pluripotent cells, inactivation of TET proteins led to the upregulation of *NODAL*, *LEFTY1*, *LEFTY2* and their downstream gene *NANOG*, whereas no difference was shown in their methylation levels (Figs. 2–E, 5G). However, *NODAL*, *LEFTY1*, *LEFTY2* and *NANOG* were rarely expressed in TKO day4 embryoids compared to WT day4 hPGCLCs, and the expression of p-SMAD2/3 was also decreased in TKO day4 embryoids, which demonstrated NODAL signaling is critical for hPGCs specification (Fig. 2C, G, Additional file 6: Fig. S6I, J).

**Fig. 3:**
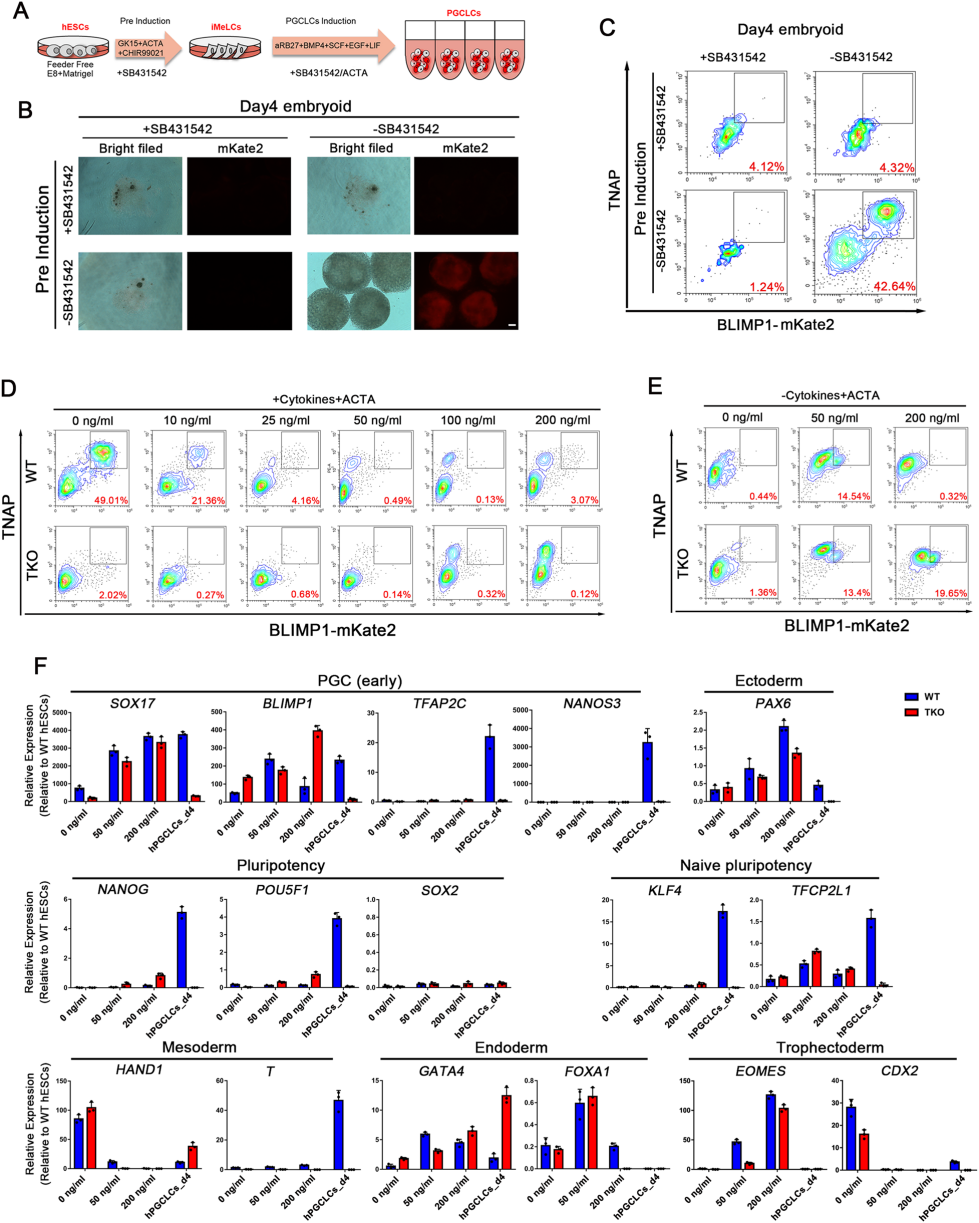
LEFTY-NODAL Signaling Pathway is Essential for hPGCLCs Differentiation. **A** Scheme of hPGC differentiation through iMeLCs in vitro; SB431524: NODAL signaling inhibitor; ACTA (Activin A): NODAL signaling stimulator; **B** Bright field (BF) and fluorescence images of day4 embryoids stimulated by cytokines and with or without SB431542 in iMeLCs or hPGCLCs induction. Scale bar = 100 μm; **C** FACS patterns show the induction efficiency of hPGCs with or without SB431542 at iMeLCs or hPGCLCs induction; **D** FACS analysis of WT and TKO hESCs in day4 hPGCLCs induction with cytokines and different concentration of Activin A; **E** FACS analysis of WT and TKO hESCs in day4 hPGCLCs induction with different concentration of Activin A and without cytokines; **F** mRNA expression was assayed in day4 embryoids treated with different concentration of Activin A without cytokines using qRT-PCR assay, the WT day4 hPGCLCs were used as positive control; n = 3 independent experiments. Data are presented as means ± s.d

Next, we used NODAL signaling inhibitor SB431542 and stimulator Activin A to investigate the role of NODAL signaling in hPGCLC induction (Fig. 3A). Our results showed that NODAL inhibitor completely blocked hPGCLC differentiation and small embryoids aggregated in day4 (Fig. 3B, C). Interestingly, adding SB431542 in the iMeLCs stage also inhibited hPGCLCs formation, because inhibited NODAL signaling in the iMeLCs stage impaired mesoderm-like cell differentiation and blocked hPGCLCs induction. This result confirms the significance of NODAL signaling in the iMeLCs phase. Both *NANOG* and *SOX17* are downstream genes of the ACTIVIN/NODAL signaling pathway and are vital for the self-renew of pluripotent cells and endoderm specification, respectively [30, 31]. Moreover, NANOG and SOX17 are essential for hPGC differentiation. When the different concentration of Activin A was added in PGCLCs induction stage, there were no TNAP/BLIMP1 double-positive cells in embryoids derived from either TKO or WT pluripotent cells (Fig. 3D), but a small number of TNAP/BLIMP1 double-positive cells were detected in embryoids induced by Activin A without other cytokines (BMP4, SCF, EGF and LIF) (Fig. 3E). However, the RT-qPCR results showed that *SOX17* and *BLIMP1* were upregulated, but other key hPGC genes such as *TFAP2C, NANOS3, NANOG* and *POU5F1* were barely expressed compared to the day4 hPGCLCs group. Notably, the embryoids also expressed endoderm, trophectoderm and ectoderm markers like *GATA4, EOMES,* and *PAX6* after Activin A treatment (Fig. 3F). These results suggest that those Activin A-induced TNAP/BLIMP1 double-positive cells were not hPGCLCs, and that ACTIVIN/NODAL signaling can activate *SOX17* only, but fails to maintain the expression of the pluripotency factor *NANOG* in hPGCLCs differentiation. Hence, we assume that TET proteins may regulate the NANOG expression in hPGC specification.

### TET proteins mediated balance between *NANOG* and *SOX17* is critical for hPGC induction

**Fig. 4:**
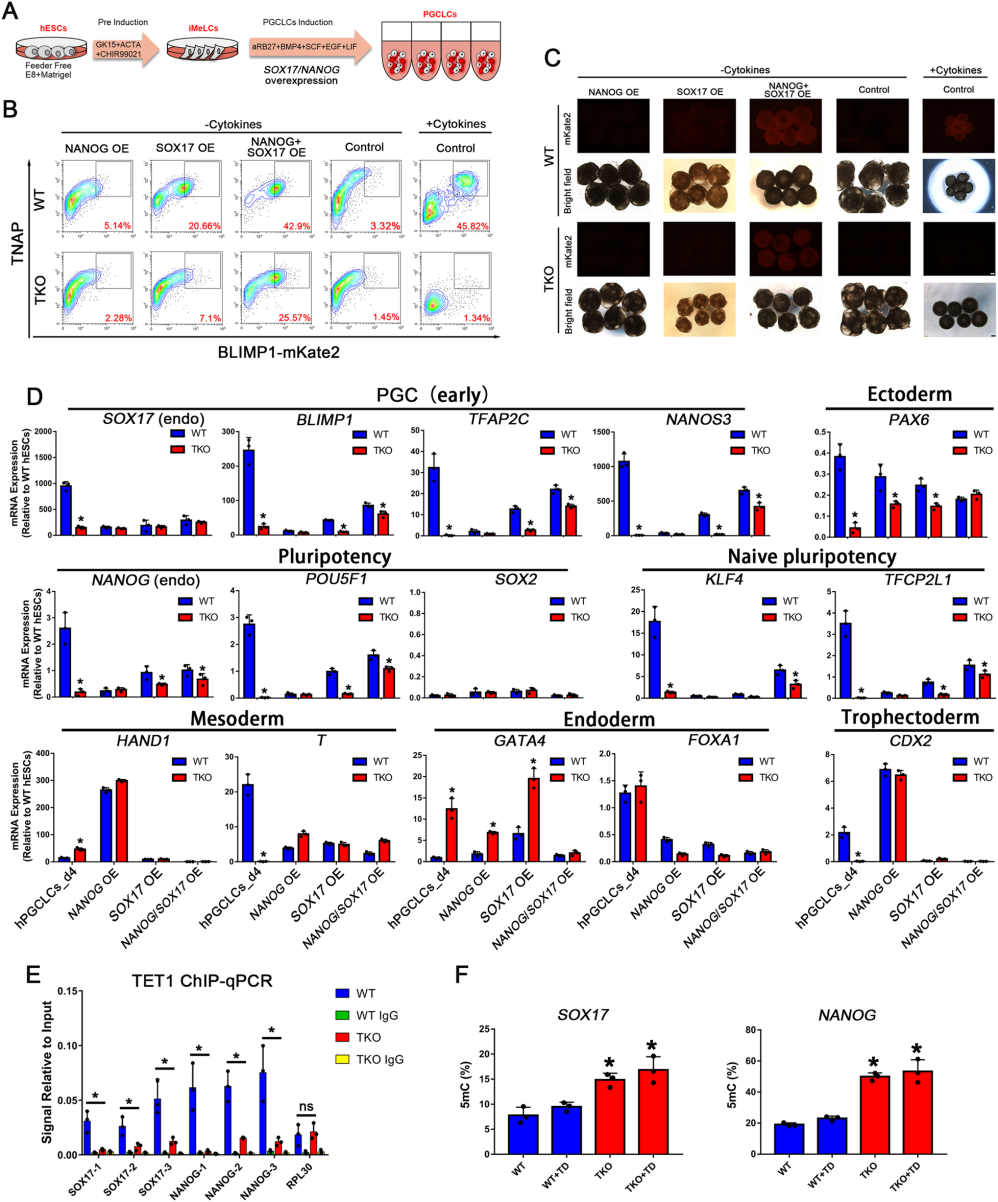
Hypermethylation of *NANOG* and *SOX17* Promoters in TET TKO hESCs Leads to a Failure of *NANOG* and *SOX17* Activation Upon hPGC Differentiation. **A** Scheme of hPGC differentiation rescue in TKO hESCs through *NANOG* and *SOX17* overexpression; **B** FACS analysis for induction of hPGCs by overexpression of *NANOG* or/and *SOX17* in WT and TKO hESCs without cytokines; **C** Bright field and fluorescence images of day 4 embryoids upon hPGC induction in **B**. Scale bar = 200 μm; **D** RT-qPCR analysis for gene expression during hPGC differentiation in day 4 embryoids by overexpression of *NANOG* or/and *SOX17* in hPGCs induction, the WT day4 hPGCLCs were used as positive control; n = 3 independent experiments. Data are presented as means ± s.d. Statistical analysis was performed by Student’s *t-*test (two-sided): * represent compared to WT group *p* < 0.05; **E** ChIP–qPCR for TET1 in WT and TKO hESCs in *NANOG* and *SOX17* promoters, RPL30 as positive control; n = 3 independent experiments. Data are presented as means ± s.d. Statistical analysis was performed by Student’s *t-*test (two-sided): *p < 0.05; **F** Analysis of the percentage of 5mC at the *NANOG* and *SOX17* promoters by Epimark with or without trimethoprim and doxycycline (TD) in day 4 embryoids upon hPGCs induction; n = 3 independent experiments. Data are presented as means ± s.d. Statistical analysis was performed by Student’s *t-*test (two-sided): * represent compared to WT group *p* < 0.05

Previous studies have demonstrated ACTIVIN/NODAL signaling and NANOG orchestrate human embryonic stem cell fate decisions and SOX17 is critical for hPGC specification [22, 32, 33]. To further distinguish the relationship between ACTIVIN/NODAL signaling and NANOG transcription in hPGCs induction, we overexpressed inducible *SOX17* and *NANOG* transgenes under the control of trimethoprim and doxycycline (TD) in hPGCLCs induction alone or together (Fig. 4A, Additional file 5: Fig. S5C). However, overexpression of NANOG individually elicited an inappreciable response after 4 days of differentiation in both WT and TKO groups, whereas SOX17 alone produces a modest response in the WT group, which is consistency with a previous study [34], but a low response in the TKO group (Fig. 4B, C). Notably, overexpression of NANOG and SOX17 together induced a strong response in the WT group with a large proportion of TNAP/BLIMP1 double-positive hPGCLCs cells and a moderate response in the TKO group, suggesting that NANOG and SOX17 act synergistically and rapidly to induce a similar response to the WT pluripotent cells treated with cytokines for hPGCLC specification. This response is preceded by downregulation of *SOX2* and upregulation of the PGC markers *NANOS3, TFAP2C*, and ‘naïve’ pluripotency genes including *KLF4* and *TFCP2L1*, as well as in vivo hPGCs. Nevertheless, endogenous expression of *NANOG* and *SOX17* was not restored as measured by RT-qPCR with primers targeting their 5’ UTR region (Fig. 4D). Global RNA-sequencing data also demonstrated that the response for hPGCLC specification induced by NANOG and SOX17 co-overexpression is similar to that induced by cytokines (Fig. 6A).

Interestingly, NANOG overexpression upregulated mesoderm marker *HAND1* and TE marker *CDX2,* while SOX17 overexpression resulted in the upregulation of endoderm marker *GATA4* (Fig. 4D). These results suggest that overexpression of *NANOG* and *SOX17* separately promoted hESCs to diverging germ layers. As reported before, NANOG could bind to the SOX17 promoter to restrain SOX17 expression in pluripotent cells and is highly expressed in hPGCs [32]. Therefore, we deduce that NANOG regulates the expression of SOX17 by binding to its promoter in hPGCs, and that the balance of NANOG and SOX17 guards the initiation of hPGC specification.

To precisely characterize the defect in differentiation potency of TKO hESCs, we also verified that TET1 could bind to the *NANOG* and *SOX17* promoters in WT hESCs by ChIP-qPCR (Fig. 4E) and further analyzed the 5mC levels of *NANOG* and *SOX17* promoters by Epimark 5mC analysis. In comparison to WT pluripotent cells, the promoters of NODAL signaling and *NANOG*/*SOX17* in TKO day4 embryoids showed much higher methylation levels (Fig. 4F, Additional file 8: S8A). Thus, the inactivation of TET proteins in hESCs causes aberrant hypermethylation of NODAL signaling genes and fails to activate *NANOG* and *SOX17* expression*,* resulting in the consequent defects in hPGC differentiation.

### De novo methylation by DNMT3B causes hypermethylation of *NANOG* and *SOX17* promoters

**Fig. 5:**
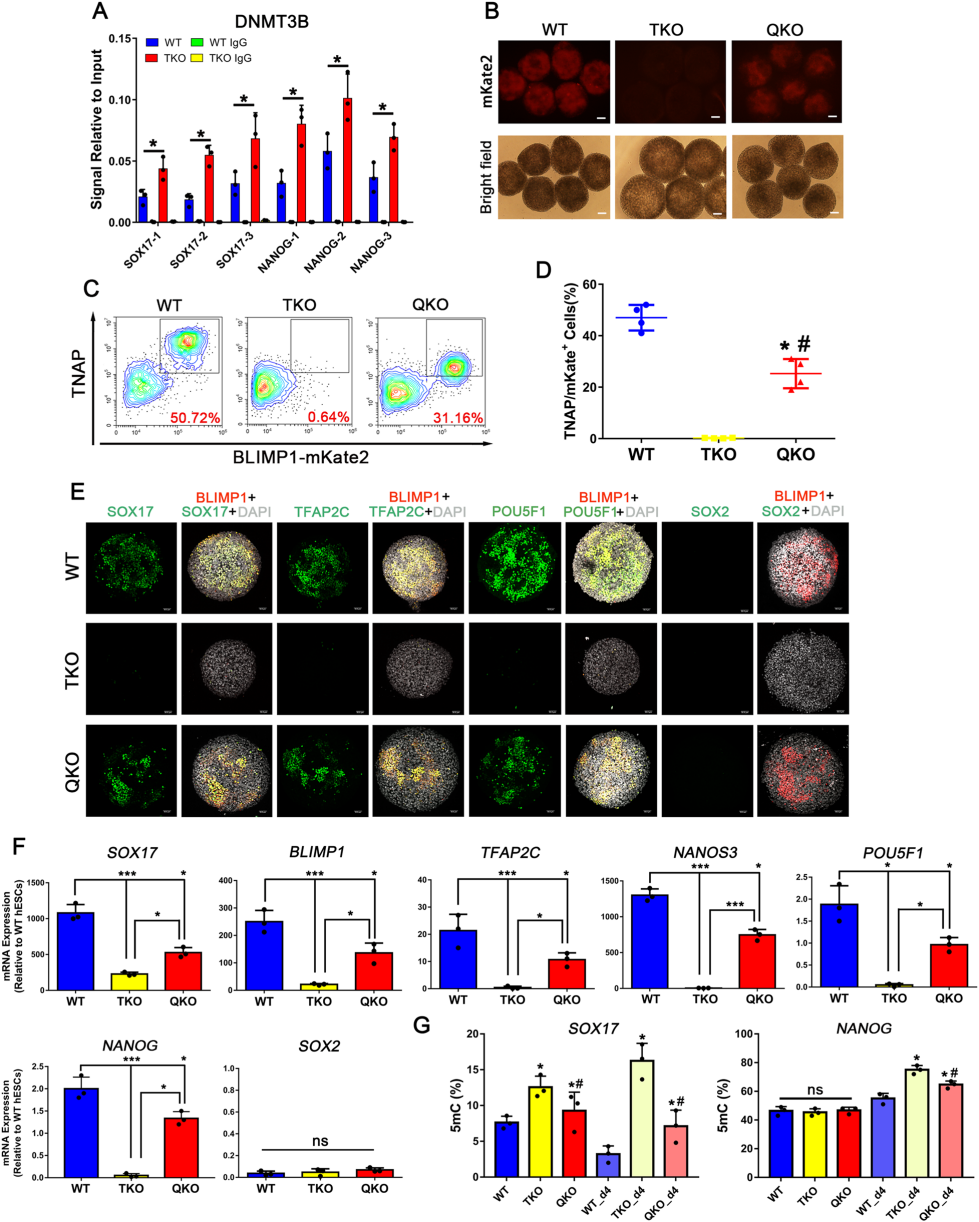
Genetic Inactivation of DNMT3B Partially Rescues the hPGCLC Differentiation Defect of TKO hESCs. **A** ChIP–qPCR for DNMT3B at the *NANOG* and *SOX17* promoters in WT and TKO hESCs; n = 3 independent experiments. Data are presented as means ± s.d. Statistical analysis was performed by Student’s *t-*test (two-sided), *p < 0.05; **B** Bright field and fluorescence images of Day 4 embryoid with BLIMP1-mKste2 reporter in WT, TKO, QKO hESCs, Scale bar = 100 μm; **C** FACS analysis for induction of hPGCs in WT, TKO, QKO hESCs; **D** Quantification of FACS at day 4 of hPGCLC induction in WT, TKO, QKO hESCs; n = 4 independent experiments. Data are presented as means ± s.d. Statistical analysis was performed by Student’s *t-*test (two-sided), * represent compared to WT group *p* < 0.05, # represent compared to TKO group *p* < 0.05; **E** Immunofluorescence of SOX17, TFAP2C, POU5F1, BLIMP1 and SOX2 at the day4 embryoid for WT, TKO and QKO cells. Scale bar = 50 μm; **F** RT-qPCR analysis for gene expression during hPGC differentiation in day 4 embryoid; n = 3 independent experiments. Data are presented as means ± s.d. Statistical analysis was performed by one-way ANOVA: *p < 0.05, **p < 0.01, ***p < 0.001; **G** Methylation analysis of the *NANOG* and *SOX17* promoters in hESCs and day 4 embryoids by Epimark; n = 3 independent experiments. Data are presented as means ± s.d. Statistical analysis was performed by one-way ANOVA: * represent compared to WT or WT_d4 group *p* < 0.05, # represent compared to TKO or TKO_d4 group *p* < 0.05

Three DNA methyltransferases, DNMT1, DNMT3A and DNMT3B, are responsible for cytosine methylation in mammals. Although *SOX17* promoter was hypermethylated in TKO hESCs, there were no differences in the expression of *DNMT* genes between WT and TKO pluripotent cells, but *DNMT1*, *DNMT3A* and *DNMT3B* were downregulated in WT d4 hPGCLCs (Additional file 6: Fig. S6A). However, ChIP-qPCR analysis showed increased binding of DNMT3B, but not DNMT1 or DNMT3A, at the *NANOG* and *SOX17* promoters in TKO hESCs as compared to WT hESCs (Fig. 5A, Additional file 6: Fig. S6B). Therefore, we inactivated the *DNMT3B* gene in TKO hESCs to further investigate whether DNMT3B is responsible for the hypermethylation of *NANOG* and *SOX17* promoters. Using the CRISPR-Cas9 technique, we generated a *TET1*, *TET2*, *TET3* and *DNMT3B* quadruple-knockout (QKO) cell line (Additional file 6: Fig. S6C–F). Like TKO hESCs, QKO hESCs still maintained normal morphology and expressed pluripotency markers, such as NANOG, SOX2, and POU5F1 (Additional file 6: Fig. S6G). There were no detectable 5hmC in QKO hESCs and no difference in 5mC levels was observed among QKO, TKO and WT hESCs (Additional file 6: Fig. S6H).

**Fig. 6:**
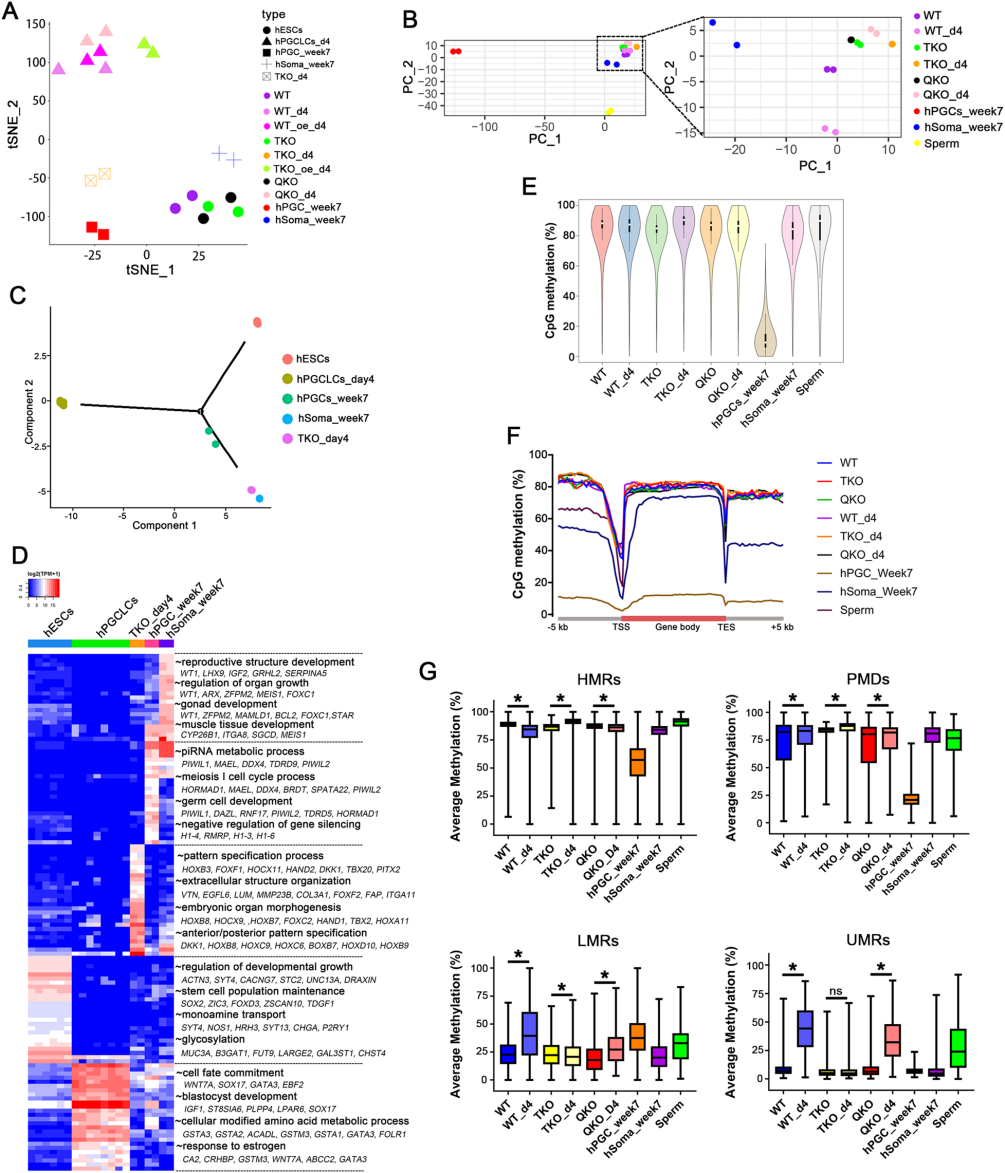
Transcriptome by RNA-seq and DNA Demethylation by Base-Resolution BS-Seq Analysis of Each Group and in vivo Data Sets. **A** tSNE plot of RNA-seq data. Color codes indicate the cell types, and shapes for cell states; **B** PCA plot of WGBS data, color codes for the cell types are indicated; **C** Pseudotime trajectory (Monocle analysis) of the cells. Cells are colored based on the predicted pseudotime; **D** Heat map of top 20 DEGs in each subpopulation estimated by ROTS. The GO functional terms and representative genes included are shown for each gene cluster; **E** Violin plots showing the distribution of CpG methylation levels in overlapped 1 kb genomic tiles of each sample, the white point indicates median; **F** Averaged CpG methylation level profiles of all genes from -5 kb upstream of the transcription start sites (TSS), through scaled gene bodies to + 5 kb downstream of transcription end sites (TES); (G) Methylation distribution according to the different segments defined by the MethylSeekR approach; n = 2 independent experiments. Data are presented as means ± s.d. Statistical analysis was performed by Student’s *t-*test (two-sided): * represent compared to hESCs group *p* < 0.05

**Fig. 7:**
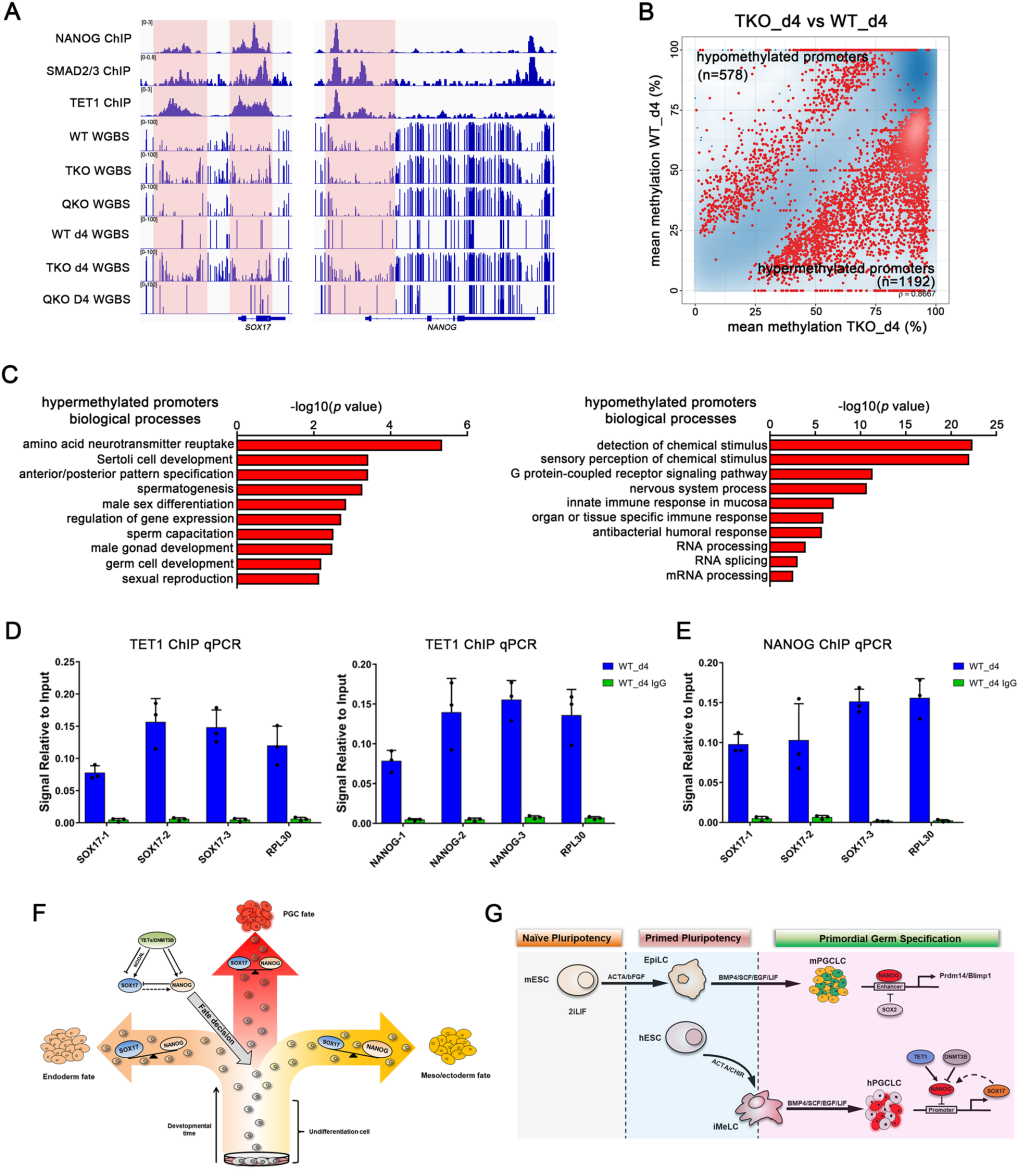
DNA methylation Controls the Balance of *NANOG* and *SOX17* in hPGC Specification. **A** NANOG, SMAD2/3, TET1 ChIP-seq binding sites and methylation profile for the *SOX17* and *NANOG* locus, red area indicated promoter region; **B** Density-scatterplot showing differentially methylated promoters in TKO day4 embryoids and WT day4 hPGCLCs; **C** GO analysis of hypermethylation and hypomethylation promoters in **B**; **D** ChIP-qPCR for TET1 in WT day4 embryoids in *NANOG* and *SOX17* promoters; **E** ChIP-qPCR for NANOG in WT day4 embryoids in *SOX17* promoters; **F** A hypothesis for epigenetic regulation of hPGC fate. The balance between NANOG and SOX17 mediated by TETs and DNMT3B guarantees hPGC specification from pluripotent cells. Overexpression of SOX17 or NANOG would compel cells to endoderm or meso/ectoderm germ layers, respectively; **G** A model illustrating the different functions of NANOG for PGC specification in mice (top) and humans (bottom). In mice, NANOG activates the expression of Blimp1 and Prdm14 by binding to their enhancers, which could be repressed by Sox2 during mPGC induction in vitro. In humans, TET1 and DNMT3B work oppositely to regulate NANOG expression during hPGC induction in vitro. And NANOG further regulates SOX17 expression by binding to its promoter

Furthermore, there was a partial rescue of the hPGC induction from QKO pluripotent cells, with about 30% of TNAP/BLIMP1 double-positive hPGCLCs, as compared to TKO pluripotent cells, and the hPGCLCs separate clearly into two populations even though the induction efficiency was still lower than WT hESCs (Fig. 5B–D). Immunofluorescence and RT-qPCR analysis also showed rescue in the expression of hPGC markers *SOX17*, *TFAP2C*, *NANOS3* and *BLIMP1*, and proper inhibition of pluripotency marker *SOX2* (Fig. 5E, F). And *NODAL*, *LEFTY1/2*, *NANOG* mRNA expression levels were rescued in QKO day4 hPGCLCs (Additional file 6: Fig. S6I). In addition, methylation levels of *NANOG* and *SOX17* promoters presented a significant reduction in QKO day4 embryoids detected by Epimark 5mC analysis and WGBS (Figs. 5G, 7A). However, the methylation level of the *NANOG* promoter was unchanged in each of the hESCs groups, implying that other epigenetic modifications or mechanisms may play a role in NANOG regulation. And the methylation levels of the *NANOG* promoter was increased in the WT_d4 group because the whole embryoid was used. Meanwhile, as a bivalent promoter (marked by H3K4me3 and H3K27me3), *SOX17* might be more sensitive to TET and DNMT3B regulation in pluripotent cells. Altogether, these results suggest that DNMT3B is the primary actor in hypermethylation of *NANOG* and *SOX17* promoters and plays a major role in the impaired hPGC differentiation in TKO hESCs. In WT hESCs, TET proteins counteract with DNMT3B to maintain the expression of *NANOG* and *SOX17* and facilitate hPGC differentiation.

### Comparison of hPGCs and hPGCLCs by RNA-seq and WGBS analysis

In order to build a comprehensive understanding of the characteristics of pluripotent cells and induced hPGCLCs in each cell line, we determined global mRNA transcription and DNA methylation profiles of each cell type during hPGCLC induction by RNA-seq and WGBS technology, and published in vivo datasets were also included in our analysis. We compared the data with gonadal hPGCs from week 7 male human embryos (Carnegie stage 18/19), which not only retain key characteristics of early hPGCs but also express later germ cell markers such as VASA and DAZL (Additional file 7: Fig. S7C).

t-Distributed Stochastic Neighbor Embedding (tSNE) analysis showed that the pluripotent cells, hPGCs_week7, hSoma_week7, day4 hPGCLCs and TKO day4 embryoids settled at five discrete positions (Fig. 6A). In particular, the day4 hPGCLCs, including the QKO day4 hPGCLCs and the *NANOG/SOX17* overexpressed TKO day4 hPGCLCs were clustered together. Unsupervised hierarchical clustering of RNA-seq transcription profiles showed that induced hPGCLCs, gonadal samples and pluripotent cells formed distinct branches. Notably, day4 TKO embryoids formed a sub-cluster with hESCs, suggesting that day4 TKO embryoids impaired hPGC differentiation capacity to form another germ layer (Additional file 7: Fig. S7A). All of the pluripotent cells were distributed together, and WT, TKO and QKO hESCs showed relatively fewer transcriptional changes compared with each other (Additional file 7: Fig. S7F–H). The WGBS PCA plot showed that the TKO and QKO hESCs were clustered together, but away from the WT hESCs. Similarly, QKO_day4 hPGCLCs distributed differentially with WT_day4 hPGCLCs, and closer with TKO_day4 embryoids (Fig. 6B). The clustered heat map of the methylation values according to the first clustered 1000 promoters showed that QKO and WT hESCs, TKO hESCs and TKO day4 embryoids formed two distinct branches (Additional file 7: Fig. S7B). These results demonstrate that even though mRNA expression patterns are consistent, the DNA methylation levels are still in diversity.

An orthogonal pseudotime analysis using the Monocle package further supported that day4 hPGCLCs and gonadal hPGCs were in different developmental branches [35], and day4 TKO embryoids and hSoma_week7 clustered together at the end of the branch (Fig. 6C). To detect DEGs that were specifically distinguished in each cell type, we performed optimized test statistic (ROTS) for the defined populations [36]. Each population was compared to the other pooled populations to find unique gene signatures and upregulated genes with an FDR < 0.001 were considered significantly differentially expressed (SDE). The top 20 SDE genes for each cell type are represented in the heatmap depicted in Fig. 6D. Next, we used the top 100 SDE genes of each population to define the gene signatures by GO analysis and described the GO terms in each subpopulation in Fig. 6D.

The global DNA methylation levels of each sample were slightly changed, but week7 gonad PGCs were going through genome-wide DNA methylation reprogramming (Fig. 6E). However, DNA methylation levels in promoters and CpG island regions were notably different, especially QKO hESCs showed a global decrease in methylation, both in promoters and CpG island (Additional file 7: Fig. S7D). The methylation patterns over genes, with low methylation at the transcription start sites (TSSs) and slightly increased levels over gene bodies, showed no obvious difference between hESCs and induced cells (Fig. 6F). Using MethylSeekR [37], we classified genome methylation regions into highly methylated regions (HMRs) and partially methylated domains (PMDs), which are in a transcriptionally repressed state, unmethylated regions (UMRs) and lowly methylated regions (LMRs), corresponding to proximal and distal regulatory sites, respectively [38, 39]. Our results showed the average methylation range of PMDs, LMRs and UMRs was increased during hPGCLCs induction from WT and QKO pluripotent cells while HMRs were decreased modestly (Fig. 6G). But in the TKO cell line PMDs and HMRs were increased during hPGCLCs induction, and LMRs were modestly decreased. This suggests that the TET and DNMT proteins dynamically regulate the methylation states of PMDs, LMRs and UMRs during hPGC specification.

Since retrotransposons take up about half of the human genome and are mainly repressed by DNA methylation [40], we also evaluated the methylation levels of major human retrotransposon classes. During the hPGCLC induction process, methylation levels of most retrotransposon loci were increased, except for Alu, SVA, and ERVK elements, which were also resisted demethylation in gonad hPGCs. Interestingly, methylation levels of all retrotransposon loci in TKO hESCs were lower than WT hESCs but were upregulated when *DNMT3B* was knocked out (Additional file 7: Fig. S7E). Therefore, we analyzed the expression of the Krüppel-associated box zinc finger protein (KRAB-ZFP) family which plays a role in restricting transposable elements activity [41, 42]. Unsupervised hierarchical clustering of all annotated KRAB-ZFPs showed the expression of many genes was changed in TKO hESCs, and DEG analysis showed *ZNF248* was downregulated in TKO versus WT hESCs, but upregulated in QKO versus TKO hESCs (Additional file 7: Fig. S7I, J). Thus, *ZNF248* may confer demethylation resistance in these retrotransposon families and be regulated by TET proteins.

Taken together, the derived hPGCLCs exhibit early-stage germ cell characteristics that are apparently en route to hPGCs, and this in vitro differentiation model provides a method to explore the epigenetic regulation mechanism at the initial stage of hPGC specification, which is otherwise not possible in vivo because postimplantation human embryos before week 4 are inaccessible to be investigated.

### Epigenetic regulation of the balance of NANOG and SOX17 during hPGC specification

A previous study reported that the ACTIVIN/NODAL signaling controls the expression of NANOG, which in turn interacts with SMAD2/3 to maintain the expression of pluripotency genes, and binds to endoderm genes to inhibit their expression [32]. We analyzed published NANOG, SMAD2/3, and TET1 ChIP-seq datasets, and found NANOG, SMAD2/3, and TET1 bind to the vicinity of PGC marker genes such as *SOX17, NANOG, BLIMP1, TFAP2C* and LETFY/NODAL signaling genes (Fig. 7A, Additional file 8: Fig. S8A). Gene-specific inspection of the methylome data revealed a significant increase in DNA methylation at the *SOX17, NANOG, BLIMP1, TFAP2C* and LETFY/NODAL signaling genes in TKO day4 embryoids (Fig. 7A, Additional file 8: Fig. S8A). Comparing TKO day4 embryoids with WT hPGCLCs, we identified 1770 differentially methylated promoters across the genome and 1192 of the promoters gained methylation after TET proteins inactivation (1192 hypermethylated promoters versus 578 hypomethylated promoters; Fig. 7B). Notably, hypermethylated promoters in TKO day4 embryoids were enriched for Sertoli cell development, anterior/posterior pattern specification and spermatogenesis; while 578 hypomethylated promoters were enriched for detection of chemical stimulus, nervous system process and RNA processing, indicating that DNA demethylation in some regions is primarily required for hPGC specification (Fig. 7C). Compared to RNA-seq results, much more changes were detected in methylation levels of promoters in different types of hESCs, and GO analysis indicated different biological processes were associated with the hypomethylated promoters (Additional file 8: Fig. S8B, C).

Further, ChIP-qPCR in day 4 hPGCLCs demonstrated that TET1 could bind to the *NANOG* and *SOX17* promoters, and NANOG could also bind to the *SOX17* promoter, which may be involved in the regulation of SOX17 expression during hPGC specification (Fig. 7D, E). Interestingly, overexpression of NANOG alone in WT hESCs is unable to promote hPGCLC differentiation. On the contrary, overexpression of SOX17 alone can induce hPGCLC differentiation in WT hESCs but not TKO hESCs (Fig. 4B). Moreover, recent research found that SOX17-TFAP2C cooperated to directly upregulate/sustain the expression of core pluripotency factors NANOG and POU5F1 during hPGCLC induction [21, 43]. But our results found that TET proteins were necessary to keep the *NANOG* promoter at a low methylation levels and that NANOG can bind to the SOX17 promoter to regulate its expression in hPGCLCs specification.

## Discussion

Global DNA demethylation is a key characteristic of mammalian PGC (and early embryo) development and allows the germ cell lineage to create a blank slate for the subsequent generation of the totipotent zygote [44]. Previous studies have focused on the transcription factors involved in the regulatory mechanisms for human germ cell development. In humans, SOX17-BLIMP1 is the central specifier of early germ cell fate, and many other key genes like *EOMES* and *TFAP2C* are critical regulators involved in the unique transcriptional program of hPGCs [21, 22, 45]. The transcriptome and DNA methylome data of hPGCs reveal the global erasure of DNA methylation in the germline genome, which is not completely correlated with the global changes in gene expression [5, 6]. However, the exact interaction between genome-wide epigenetic reprogramming and transcriptome regulatory networks during hPGC specification remains unclear and debatable.

The genome-wide DNA demethylation occurs by TET-mediated hydroxymethylation of 5mC to 5hmC. Tet1and Tet1/2 double mutant mice are viable, fertile, and grossly normal, though some mutant mice have a slightly smaller body size at birth [46, 47]. And different from humans, Tet2 depletion resulted in a much greater decrease in genomic 5hmC levels than Tet1 depletion in mESCs [48]. Inactivation of all three *Tet* genes diminished the expression of the *Lefty1* and *Lefty2* genes and hyperactive of Nodal signaling, resulting in impaired gastrulation including primitive streak patterning defects [27]. Different from mice, knockout of three *TET* genes in hESCs downregulated *LEFTY1*, *LEFTY2*, as well as *NODAL* and their target gene *SOX17* when hESCs were induced into hPGCLCs. As a result, the imbalance of NANOG and SOX17 impairs the ability of hESCs to differentiate into hPGCLCs in vitro. The different roles of TET proteins in mice and humans illustrate that species diversity limits the application of animal research to humans directly.

In mice, dose-dependent Nodal/Smad functional activities are known to be required for A–P and left–right axis patterning during early embryo development. The nodal signaling functions in vivo to promote optimal levels of Nanog during pre-mPGC development and also to spatially restrict the mPGC niche [29]. In pluripotent cells, ACTIVIV/NODAL signaling controls the expression of the key pluripotency factor NANOG, which in turn interacts with SMAD2/3 to maintain the expression of pluripotency genes. In addition, NANOG and SMAD2/3 can bind to the promoters of endoderm genes like SOX17 without inducing their expression, suggesting the action of other factors capable of blocking the expression of such genes in hESCs [32]. The expression of NANOG is upregulated in hPGC, but ACTIVIV/NODAL signaling only activates SOX17 rather than NANOG in hPGCLCs induction. And the ectopic expression of NANOG in hESCs blocked hPGCLCs progression. Conversely, SOX17 is not critical for mPGC differentiation, and overexpression of Nanog along can induce PGCLCs in mouse EpiLCs (epiblast-like cells) [49], which illustrates that NANOG plays different roles in human and mouse PGC specification (Fig. 7F, G).

Our previous study found that only 40–42 h of mesoderm-like cells induction could induce hPGCLCs with highly efficient, and other studies demonstrated that the Wnt and NODAL signaling dose and window time were critical for hPGC differentiation [26, 50]. Therefore, adding Activin A in the hPGC induction stage forced cell fate to endoderm (Fig. 3F, expression highly SOX17 and BLIMP1 but no expression of PGC marker TFAP2C and NANOS3). Because Activin A in TKO cell upregulated SOX17, rather than NANOG (regulated by TET proteins) to establish transcription factor loop to acquire PGC fate. A recent study has reported that overexpression of SOX17 alone or together with BLIMP1 can induce an hPGCLC-like phenotype in 4i hESCs in the absence of cytokines [34]. Similarly, our result showed overexpression of SOX17 alone or in combination with NANOG was also sufficient for hPGCLCs induction in the absence of cytokines in WT hECCs, but the efficiency declined significantly when all three *TET* genes were inactivated. Moreover, disruption of DNMT3B in the TET-deficient hESCs could obviously restore LEFTY/NODAL signaling and hPGCLC specification. Therefore, our study provides a key insight that epigenetic regulation is closely involved in cell fate determination during hPGC specification. Especially, TET proteins and the de novo methyltransferases DNMT3B work competitively to balance the methylation levels of gene regulatory elements and to maintain the accuracy of transcriptional networks and signaling for early human germline development. Our results proposed that TET proteins control the LEFTY/NODAL signaling to activate SOX17 and maintain the hypomethylation state of the SOX17 promoter. Meanwhile, the expression of NANOG was maintained by TET proteins to keep cells in a pluripotent state, and NANOG simultaneously binds to the SOX17 promoter to regulate its expression and facilitate PGC progression instead of differentiation into endoderm cells. Hence, NANOG is an important transcription factor that maintains a specific cellular state during periods of gene expression turbulence. This balance between NANOG and SOX17 regulated by TET proteins is pivotal for hPGC specification, which is different from the mouse model.

Interestingly, knockout of TET genes in hESCs contributed to aberrant hypermethylation of *NANOG* and *SOX17* promoters, but the DNA methylation levels were reduced and the hPGC induction was partially rescued in DNMT3B inactivated QKO hESCs. Thus, this suggests that DNMT3B plays a major role in the hypermethylation phenotype of TKO hESCs, and this is inconsistent with the high overall fidelity of DNMT1 for maintaining DNA methylation patterns. Because of the divergent substrate preference between DNMT3A and DNMT3B [51, 52], DNMT3B may exhibit stronger activity than DNMT3A at *NANOG* and *SOX17* promoters, even though they share largely overlapping targets. Notably, even though the inactivation of TET proteins does not cause remarkable changes in the hESCs transcriptome, it impairs the differentiation potential of hESCs. Here, we suppose that TETs-mediated epigenetic regulation affects the differentiation potential of hESCs for the human germline without immediate impacts on gene expression.

In conclusion, the dynamic and balanced expression of NANOG and SOX17 controlled by DNA methylation and demethylation plays a crucial role in hPGC specification. In the absence of TET proteins, aberrant methylation of PGC regulators leads to impaired LEFTY/NODAL signaling and turbulence in *NANOG* and *SOX17* expression, which compels hESCs to differentiate into diverging germ layers and ultimately results in hPGCLCs specification failure. Thus, TET proteins and the de novo methyltransferase DNMT3B work competitively to balance the methylation levels of gene regulatory elements and maintain the accuracy of transcriptional networks and signaling for early human germline development. Our study presents a new advance in the epigenetic regulation of early human germline development which was different from that discovered in mice. A recent study in mouse models reveals an unexpected role for TET1 in maintaining but not driving DNA demethylation in gonadal mPGCs development [53]. Based on these findings, the epigenetic mechanism regulate key signaling pathways and gene networks in early germline development. It is meaningful to take advantage of the locus-specific epigenome editing techniques to directly explore the function of epigenetic changes with in vitro models. Therefore, further research should aim at establishing better methods to promote the differentiation of hPGCLCs into more mature germ cells, or even to recapitulate complete gametogenesis in vitro, especially with epigenetic reprogramming.

## Materials and methods

### hESC culture

BLIMP1-mKate H1 ESC line was established previously [26]. The ESCs were maintained under a feeder-free condition in mTeSR1 (Stem Cell Technologies, 85850) or TeSR-E8 medium (Stem Cell Technologies, 05990) on Matrigel (Corning, 356234)-coated cell culture plates. Cultures were passaged at a 1:10 to 1:20 split ratio every 4–6 days using 0.5 mM EDTA/PBS (Thermo Fisher, AM9260G). 10 μM Rho-associated protein kinase (ROCK) inhibitor Y-27632 (Selleck Chemicals, S1049) was added into the culture medium when passaging or thawing cells.

### Generation of mutant lines

CRISPR gRNAs for *TET1*, *TET2*, *TET3* and *DNMT3B* are listed in Additional file 9: Tables S1–S7, and the gRNAs were cloned into pX330-U6-Chimeric_BB-CBh-hSpCas9. For transfection, 80% confluent cells were dissociated into single cells using Accutase (Thermo Fisher, 00-4555-56). 1 × 10^6^ cells were plated in Matrigel coated 35-mm dish and transfected with 1 μg of the CRISPR plasmid in 100 μl of OptiMEM (Thermo Fisher, 31,985,088) using 3 μl of FuGENE 6 (Promega, E2691). 2 μM Thiazovivin (Selleck Chemicals, S1459) was added in culture medium to improve survival rates of cells. Another transfection was performed 24 h later to improve the transfection rate. 24 h after the two rounds of transfection, hESCs were dissociated into single cells and re-plated at ~ 800 cells per 10-cm dish and CloneR (Stem Cell Technologies, 05,889) was added to the culture medium. 8 ~ 10 days later, the single cell derived colonies were dissociated by Collagenase IV (Stem Cell Technologies, 07,909) and manually picked individually into 48-well plates for amplification. Colonies were analyzed by Sanger sequencing at the three *TET* genes and *DNMT3B* for the presence of mutations. The cell pellets were suspended in 20 μl of water, and 2 μl of suspended cells were used for PCR amplification of the target site with the KOD FX (TOYOBO, KFX-101) kit according to the manufacturer’s protocol. The primers are listed in Additional file 10: Tables. Clonal cell lines carrying the desired mutations were amplified and frozen. WT cell lines were used as passage-matched controls for methylation analysis and differentiation.

### Induction of hPGCLCs

Induction of hPGCLCs was performed according to a previous report [26]. Briefly, hESCs were dissociated with 0.5 mM EDTA/PBS and 1 × 10^6^ cells per well were plated on Matrigel coated 6-well plates in GK15 medium (G-MEM [Thermo Fisher, 11,710–035], 15% KSR [Thermo Fisher, 10,828–028], 0.1 mM NEAA [Thermo Fisher, 11,140–050], 2 mM L-glutamine [Thermo Fisher, 35,050–061], 1 mM sodium pyruvate [Thermo Fisher, 11,360–070], 0.1 mM 2-mercaptoethanol [Sigma, M3148], 3 μM CHIR99021 [Selleck Chemicals, S2745], 50 ng/ml Activin A [PEPRO TECH, 120-14E] and 10 μM ROCK inhibitor) for pre-induction. After 40 ~ 42 h of pre-induction, the cells were dissociated with Accutase (Thermo Fisher, A1110501) and plated into ultra-low cell attachment U-bottom 96-well plates (Corning, 7007) at a density of 2,000–4,000 cells per well to form embryoid bodies in 200 μl of aRB27 induction medium (Advanced RPMI 1640 [Thermo Fisher, 12,633–012], 1% B-27 supplement [Thermo Fisher, 17,504–044], 0.1 mM NEAA, 2 mM L-glutamine, 500 ng/ml BMP4 (R&D Systems, 314-BP-050), 10 ng/ml human LIF (R&D Systems, 7734-LF-100), 100 ng/ml SCF (R&D Systems, 255-SC-050), 50 ng/ml EGF (R&D Systems, 236-EG-200), and 10 μM ROCK inhibitor (Selleck, S1049) or 10 μM SB431542 (Selleck, S1067).

### Flow cytometry analysis

The floating aggregates were dissociated with 0.05% Trypsin–EDTA/PBS for 15 min at 37 °C. After washing with PBS, the cell suspension was filtered by a cell strainer to remove cell clumps and then subjected to centrifugation. To analyze hPGCLCs or hESCs with cell surface markers, the dissociated cells were stained with FITC-conjugated anti-human/mouse TNAP, PE-conjugated anti-TRA-1–60, or FITC-conjugated anti-SSEA-4. Intracellular staining was performed using a BD kit (BD, 560,589) according to the manufacturer’s instructions with PerCP-Cy™5.5 Mouse anti-Oct3/4, PE Mouse anti-human NANOG or Alexa Fluor® 647 Mouse anti-SOX2. The primary antibodies used in this study are listed in Additional file 10: Tables. The stained cells were resuspended in PBS and analyzed (Beckman, DxFLEX) or sorted (BD, FACS Aria III) with a flow cytometer.

### Quantitative RT-PCR

Total RNA was extracted using TRIzol reagent (Thermo Fisher, AM9738) or MicroElute Total RNA Kit (OMEGA, R6831-01). cDNA was synthesized using Reverse Transcription Kit (Takara, RR047A). The qRT-PCR was performed using SYBR Premix Ex Taq II (Takara). The primers used are shown in Additional file 9: Tables. Values normalized to GAPDH and relative to control samples are shown. Error bars are mean ± SD from three independent experiments.

### Immunofluorescence

Day 4 embryoid bodies induced from hESCs were fixed in 4% paraformaldehyde at room temperature for 15 min. After washing and permeabilization for 10 min with the Wash Buffer (0.01% Triton X-100 and 1.0% BSA in PBS), the aggregates were incubated with primary antibodies in Wash Buffer at 4℃ overnight. Then the aggregates were washed three times and incubated with secondary antibodies at room temperature for one hour. After washing for three times, the aggregates were incubated with DAPI for 5 min at room temperature, and images were captured with a confocal microscope (Leica) and processed with Leica software. The primary and secondary antibodies used are listed in Additional file 10: Tables.

### Plasmid construction and gene introduction

The plasmid for the doxycycline-induced overexpression was constructed based on the Gateway System (Thermo Fisher Scientific). The sequences of human *SOX17* cDNA were amplified from pENTR-SOX17 (Vigene Bioscience, CH803137), and human *NANOG* cDNA was amplified from pENTR-NANOG (Vigene Bioscience, CH832818) and DHFR was amplified by PCR from pBMN-DHFR-YFP (Addgene, #29,325). The PCR products were first cloned into pENTR1A no ccDB (Adegene, #17,398) by Gibson Assembly Mix (NEB, #E2611) according to the manufacturer’s protocol and were then recombined into the pInducer20 vector (Addgene, #44,012) or pLEX_307 (Addgene, #41,392) with LR Clonase Enzyme Mix (Thermo Fisher, 11,791,020). The destination vector was designed as shown in Additional file 5: Fig. S5C. All sgRNA were cloned into the px330 vector (Addgene, #42,230), sgRNAs used for the construction are shown in Additional file 9: Tables.

1 × 10^6^ hESCs were then infected by the lentiviruses. 100 μg/ml Geneticin (Thermo Fischer, 10,131,027), and 0.25 μg/ml puromycin (Thermo Fisher, A1113803), were added 7 ~ 10 days after the infection. The induction of the transgene upon 1 μg/ml doxycycline (Sigma-Aldrich, D9891) and 10 μM Trimethoprim (TMP) (Sigma-Aldrich, T7883) administration of the selected hESC clones was assessed at hPGCLC induction period.

### ChIP-qPCR

ChIP-qPCR was performed using the SimpleChIP Plus Enzymatic Chromatin IP kit (Cell Signaling Technology, 9003S) according to the manufacturer’s protocols. The antibodies and primers used for ChIP and qPCR are provided in Additional file 9: Tables.

### Epimark analysis

Day4 embryoids were disaggregated using Accutase, and genomic DNA was extracted using the DNeasy Blood & Tissue kit (Qiagen, 69,504) following the manufacturer’s guidelines. Epimark analysis was performed using the EpiMark 5mC Analysis Kit (NEB, E3317S) according to the manufacturer’s protocols. The primers used for qPCR are provided in Additional file 9: Tables.

### Dot Blot

Dot Blot assays were performed according to published protocols [54], Genomic DNA was diluted to 100 ng/μl in 20 μl total volume. 5 μl of 0.5 M NaOH was added to each sample and incubated at 99 °C for 5 min. Samples were neutralized with 2.5 μl of 6.6 M Ammonium Acetate. 2.75 μl of each mixture was spotted on a nitrocellulose membrane and allowed to air dry. The membrane was baked for 2 h at 80 °C and then incubated in blocking buffer for 2 h at RT. 5hmC and 5mC antibody diluted in blocking buffer was added to the membrane and incubated overnight at 4 °C. The membrane was washed in PBS with 0.1% Tween 20, 3 times for 15 min each and then incubated with a second antibody in blocking buffer for 2 h at RT. The membrane was washed again 3 times in PBS with 0.1% Tween 20. Thermo Scientific Pierce ECL Western Substrate (Thermofisher, 32,106) was used for detection. The original dot blot results were in Additional material.

### Western Blot

Whole-cell extracts were in lysis buffer composed of 50 mM Tris–HCl (pH 7.5), 0.15 M NaCl, 0.1% SDS, 1% Triton X-100, 1% sodium deoxycholate and protease inhibitor cocktail (Sangon Biotech, C600386). After electrophoresis, proteins were transferred to nitrocellulose membranes. Membranes were incubated in the western blocking reagent (TBST with 5% non-fat milk) for 1 h at RT. Antibody diluted in blocking buffer was added to the membrane and incubated overnight at 4 °C. The membrane was washed in TBST, 3 times for 15 min each and then incubated with second antibody in blocking buffer for 1 h at RT. The membrane was washed again 3 times in TBST. Thermo Scientific Pierce ECL Western Substrate (Thermofisher, 32,106) was used for detection. The original western blot results were in Additional material.

### RNA-seq analysis

For RNA-Seq data in Fig. 6, hESCs mRNA was purified from total RNA using oligo(dT)-attached magnetic beads. For hPGCLCs from day 4 embryoids, TNAP and BLIMP1-mKate2-double positive cells were sorted. Before mapping, reads were quality-trimmed (Q > 25) and the adaptor sequence was removed using Trim-Galore v0.6.4. Reads were mapped to the human reference genome (GRCh37/hg19) by HISAT2 v7.5.0. Read counts were derived from the feature Counts v2.0.1 with default parameters. The R Bioconductor DESeq2 package v1.28.1 was used to normalize counts per Ref Seq transcripts to evaluate the differential expression. Before clustering and principal component analysis, the transcripts with the 10% lowest average expression were removed, and the gene expression data matrix was centered and scaled. Principal component analysis was performed by the R Bioconductor Seurat package v3.2.0. Trajectory analysis was performed by the R Bioconductor Monocle2. Gene ontology analysis was performed by the R Bioconductor ClusterProfiler package v3.16.1.

### Comparison of gene expression between humans and mice

In order to compare human genes with mice, all transcript annotations for humans (ref_GRCh37, including all exon data) were converted to the mouse genome (ref_GRCm38) coordinates using the LiftOver utility. The chain files used for LiftOver were obtained from the UCSC. Then, the human gene annotations in GRCm38 coordinates were compared with ref_ GRCm38 and followed by a search for the corresponding GRCm38 transcripts. We performed the same procedure on ref_ GRCm38, in which transcript annotations in ref_ GRCm38 were converted to hg19 coordinates using LiftOver followed by a search for the corresponding human transcripts. A total of 17,932 genes were identified using the two types of comparison, and these were set as the confidently matched genes between human (24,669) and mouse (20,370) genes.

### WGBS

For WGBS, 1 μg of genomic DNA was sheared using an E220 focused ultrasonicator (Covaris) into 250- to 350-bp fragments. After end repair and add A-Tailing to the 3’ end, DNA libraries were denatured and treated with bisulfite for 30 min at 65 °C. ssDNA was purified with Methylation-Gold kit (ZYMO). Agarose gel electrophoresis was performed on the ligation product, and fragments ranging from 320 to 420 bp were selected. The gel was purified with QIAquick Gel Extraction kit (QIAGEN). The amplified libraries were quantified and sequencing on an Illumina sequencing system. For PBAT-WGBS, sorted cells were stored in the lysis buffer and sent to BGI in dry ice, sequencing on an Illumina sequencing system. WGBS data were aligned to the bisulfite-converted hg19 reference genome using Bismark v0.22.3. We extracted methylation status with the bismark_ methylation_extractor script in Bismark. Only CpGs with at least three reads covering them were used for downstream analysis. Downstream data analysis was performed by the R Bioconductor RnBeads v2.7.0 in default parameter. RepeatMasker annotations for the human reference genome were obtained from the UCSC Table Browser. Sequencing datasets analyzed in this study are provided in Additional file 9: Tables.

### Statistical analysis

Data are presented as means ± s.d. and were derived from at least three independent experiments. Data on replicates (n) are given in Figure legends. Statistical analysis was performed using the two side Student’s *t*-test (comparing two groups) or one-way-comparison ANOVA (comparing multiple groups against one group).

## Supplementary Information

**Additional file 1: Fig. S1.** Generation of *TET* Gene Knockout hESC Lines, Related to Fig. 1. (A) Design of the CRISPR targets for *TET* genes, using gRNAs (red arrows) that target the sequences corresponding to the beginning of the catalytic domain in TET1, TET2 and TET3; (B) The efficiency for the homozygous knockouts of the *TET* alleles. The knockouts (KO) were confirmed as bi-allelic frame-shift nonsense mutations. The others include wild-types or heterozygous mutants, or alleles with deletions/insertions of 3 × N base pairs; (C) The DNA sequences of both alleles for the indicated knockout lines. Red letters indicate the positions of the guide RNAs. del: deletion; ins: insertion; (D) Sanger sequencing of TKO hESCs in *TET* gene target locus.

**Additional file 2: Fig. S2.** The 5hmC/5mC Levels and Growth Curves in *TET* Gene Knockout hESCs, Related to Fig. 1. (A) Analysis of 5hmC and 5mC levels in each cell line by dot blot; (B) Analysis of 5hmC and 5mC levels in WT, TKO and *TET2/3* DKO *TET1* heterozygote cell lines by dot blot; (C) Growth curves for WT and TKO hESCs. Error bars indicate mean ± s.d. from three independent biological replicates.

**Additional file 3: Fig. S3.** hPGCLCs Differentiation is Sensitive to TET Gene Dosage, Related to Fig. 1. (A) Bright field and fluorescence images of day 4 embryoids with BLIMP1-mKste2 reporter in each cell line, Scale bar = 200 μm; (B) Representative FACS plots of TNAP/BLIMP1 positive cells at day 4 of hPGCLC differentiation for TET-knockout mutants; (C) Immunofluorescence of SOX17, TFAP2C, POU5F1, BLIMP1 and SOX2 at the day4 embryoids for TET-knockout mutants. Scale bar = 50 μm; (D) Quantification of FACS at day 4 of hPGCLC induction in TET-knockout mutants; n = 4 independent experiments. Data are presented as means ± s.d. Statistical analysis was performed by Student’s *t-*test (two-sided), compared to WT group *p < 0.05, ***p < 0.001.

**Additional file 4: Fig. S4.** RT-qPCR analysis for *SOX17, BLIMP1, TFAP2C, NANOS3, POU5F1, SOX2* during hPGC differentiation in day 4 embryoids, Related to Fig. 1; n = 3 independent experiments. Data are presented as means ± s.d. Statistical analysis was performed by Student’s *t-*test (two-sided), compared to WT group, *p < 0.05, **p < 0.01, ***p < 0.001, Related to Fig. 1.

**Additional file 5: Fig. S5.** *TET* Gene Knockout Shows No Effect on iMeLCs Induction, Related to Fig. 1. (A) Bright filed of iMeLCs induction from WT and TKO hESCs (42 h); (B) RT-qPCR analysis for each gene during iMeLCs differentiation in 42 h; n = 3 independent experiments. Data are presented as means ± s.d; (C) Vectors for overexpression of DOX-inducible *SOX17*, and TMP-inducible *NANOG* transgenes in BLIMP1–mKate2 reporter hESCs.

**Additional file 6: Fig. S6.** DNMT3B Deletion Partially Rescues Hypermethylation of *NANOG* and *SOX17* Promoters in TKO hESCs, Related to Fig. 5. (A) RNA-seq analysis of *DNMT1*, *DNMT3A* and *DNMT3B* in WT hESCs, TKO hESCs, WT d4 PGCLCs and TKO day4 embryoids; n = 2 independent experiments. Data are presented as means ± s.d. Statistical analysis was performed by Student’s *t-*test (two-sided); (B) ChIP–qPCR for DNMT1 and DNMT3A at the *SOX17* and *NANOG* promoters in WT and TKO hESCs, RPL30 was used as positive control supplied in ChIP kit; n = 3 independent experiments. Data are presented as means ± s.d. Statistical analysis was performed by Student’s *t-*test (two-sided); (C) Design of the CRISPR targets for *DNMT3B* genes, using gRNAs (red arrows) that target the sequences corresponding to the beginning of the catalytic domain in DNMT3B; (D) The DNA sequences of both alleles for the indicated knockout lines. Red letters indicate the positions of the guide RNAs. del: deletion; ins: insertion; (E) The efficiency for the homozygous knockouts of the *TET* alleles. The knockouts were confirmed as bi-allelic frame-shift nonsense mutations. The others include wild-types or heterozygous mutants, or alleles with deletions/insertions of 3 × N base pairs; (F) Western blots showing DNMT3B expression level in WT, TKO, QKO hESCs; (G) Left, A phase-contrast image of QKO hESCs. Scale bar = 100 μm. Right, FACS analysis for POU5F1, SOX2, NANOG, TRA-1–60, and SSEA-4 expression in QKO hESCs; (H) Analysis of 5hmC and 5mC levels in WT, TKO, QKO hESCs by dot blot; (I) mRNA expression was assayed in day4 embryoids using qRT-PCR assay; n = 3 independent experiments. Data are presented as means ± s.d.; Statistical analysis was performed by Student’s *t-*test (two-sided), * represent compared to WT group *p* < 0.05, # represent compared to TKO group *p* < 0.05, & represent compared to WT_d4 group *p* < 0.05, $ represent compared to TKO_d4 group *p* < 0.05; (J) Western blots showing SMAD2/3 and p-SMAD2/3 expression level in WT, TKO, QKO hESCs and day4 embryoids.

**Additional file 7: Fig. S7.** Transcription and DNA methylation Profile of Each Cell Lines, Related to Fig. 6. (A) Unsupervised hierarchical clustering of the transcriptomes (two independent experiments) of each sample, AU (Approximately Unbiased) *p*-value and BP (Bootstrap Probability) value; (B) Hierarchical clustering on the top changed 1000 promoters by RnBeads; (C) Heat map of key PGC-associated genes (early and late), pluripotency, mesoderm, endoderm, and gonadal somatic (Soma) markers; (D) Violin plots showing distribution of CpG methylation in gene region, promoter and CpG island, white point indicates median; (E) Violin plots showing distribution of CpG methylation in major human repetitive elements classes and families, white point indicates median; (F) Volcano plot of RNA-seq data illustrating transcriptional changes in TKO as compared to WT hESCs; n = 2 independent experiments; (G) Volcano plot of RNA-seq data illustrating transcriptional changes in QKO as compared to WT hESCs; n = 2 independent experiments; (H) Volcano plot of RNA-seq data illustrating transcriptional changes in QKO as compared to TKO hESCs; n = 2 independent experiments; (I) Unsupervised hierarchical clustering of KRAB-ZFPs expressions in WT, TKO, QKO hESCs; (J) Volcano plot of KRAB-ZFPs expressions illustrating transcriptional changes in TKO as compared to WT hESCs, and QKO as compared to TKO hESCs; n = 2 independent experiments.

**Additional file 8: Fig. S8.** Differential Methylation Promoters and GO Analysis, Related to Fig. 7. (A) NANOG, SMAD2/3, TET1 binding sites and methylation profile for the *BLIMP1, TFAP2C, NODAL, LEFTY1* and *LEFTY2* locus, the red area indicated promoter region; (B) Density-scatterplot showing differentially methylated promoters in each cell line; (C) GO analysis of hypomethylation promoters in Fig. S8B.

**Additional file 9: Tables S1–S7.**

**Additional file 10: Table S8.**

## Data Availability

The China National Center for Bioinformation accession number for the RNA-seq and WGBS data reported in this paper is HRA000405.
