# Supplementary figures and images for: The balance between NANOG and SOX17 mediated by TET proteins regulates specification of human primordial germ cell fate
Source: Cell Biosci. 2022 Nov 4;12:181. doi: 10.1186/s13578-022-00917-0 (PMC9636699; doi:10.1186/s13578-022-00917-0)

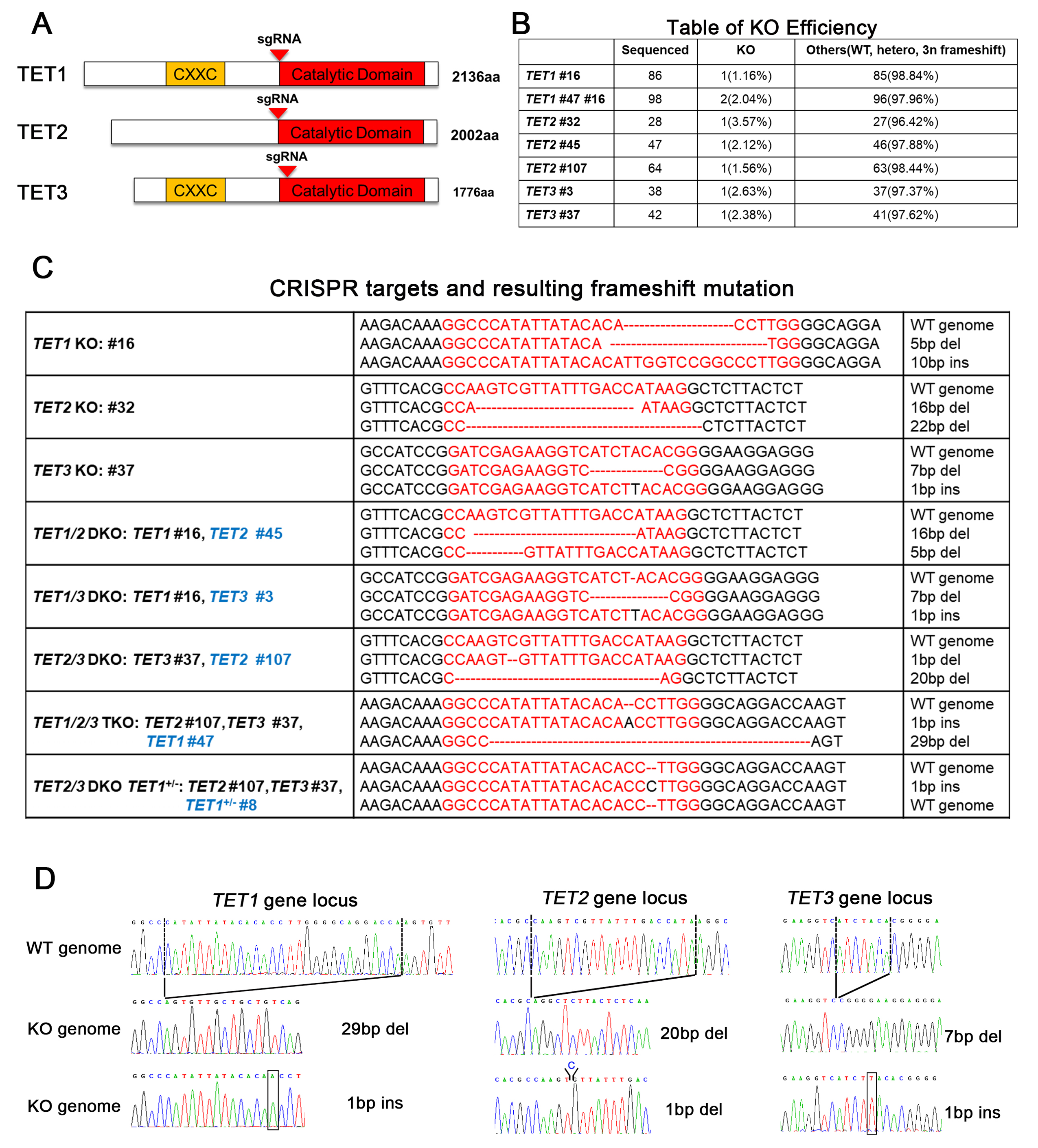

Supplement: Supplementary file 1 — Additional file 1: Fig. S1. Generation of TET Gene Knockout hESC Lines, Related to Fig. 1. (A) Design of the CRISPR targets for TET genes, using gRNAs (red arrows) that target the sequences corresponding to the beginning of the catalytic domain in TET1, TET2 and TET3; (B) The efficiency for the homozygous knockouts of the TET alleles. The knockouts (KO) were confirmed as bi-allelic frame-shift nonsense mutations. The others include wild-types or heterozygous mutants, or alleles with deletions/insertions of 3 × N base pairs; (C) The DNA sequences of both alleles for the indicated knockout lines. Red letters indicate the positions of the guide RNAs. del: deletion; ins: insertion; (D) Sanger sequencing of TKO hESCs in TET gene target locus. [file 13578_2022_917_MOESM1_ESM.tif]

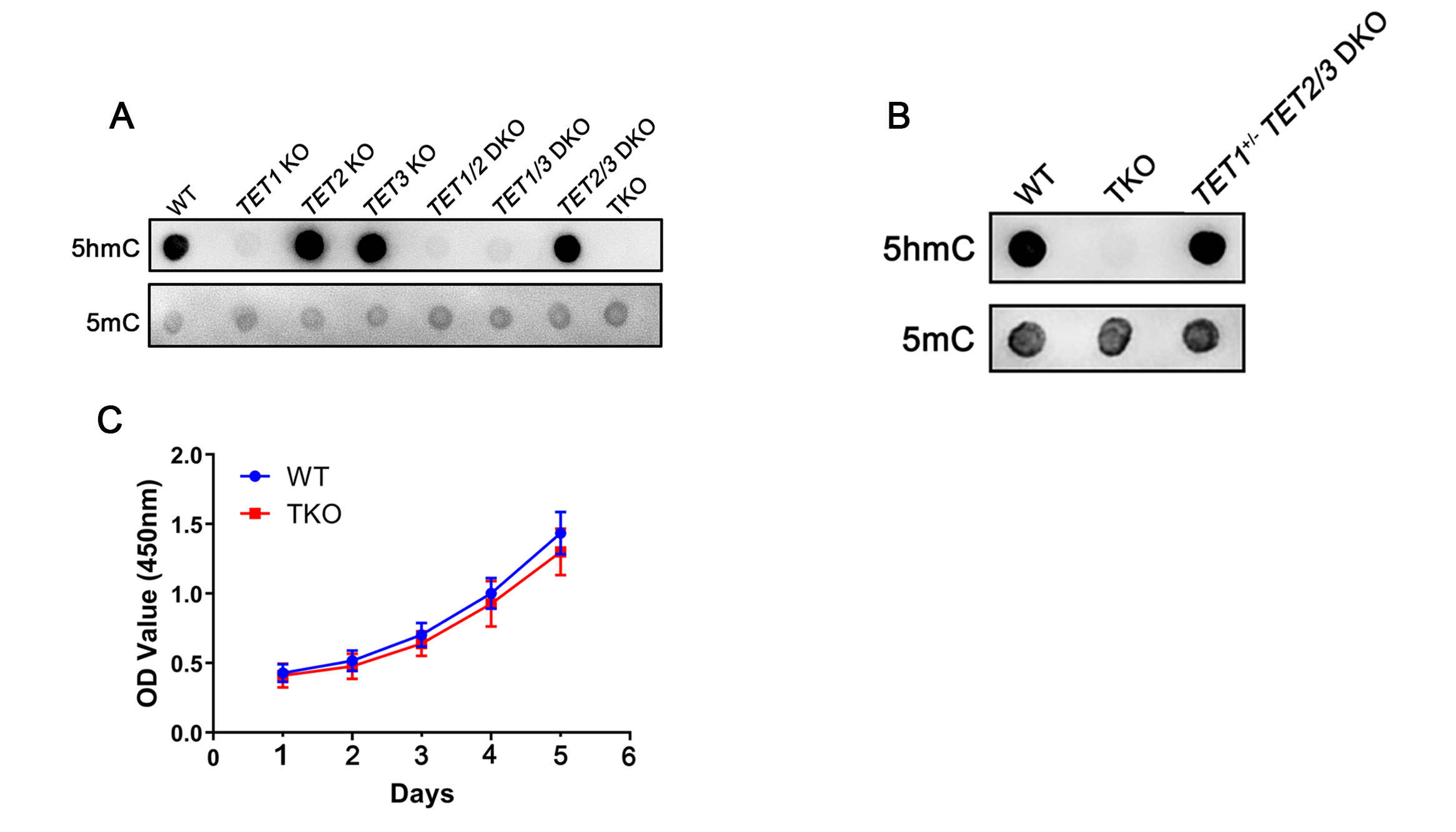

Supplement: Supplementary file 2 — Additional file 2: Fig. S2. The 5hmC/5mC Levels and Growth Curves in TET Gene Knockout hESCs, Related to Fig. 1. (A) Analysis of 5hmC and 5mC levels in each cell line by dot blot; (B) Analysis of 5hmC and 5mC levels in WT, TKO and TET2/3 DKO TET1 heterozygote cell lines by dot blot; (C) Growth curves for WT and TKO hESCs. Error bars indicate mean ± s.d. from three independent biological replicates. [file 13578_2022_917_MOESM2_ESM.tif]

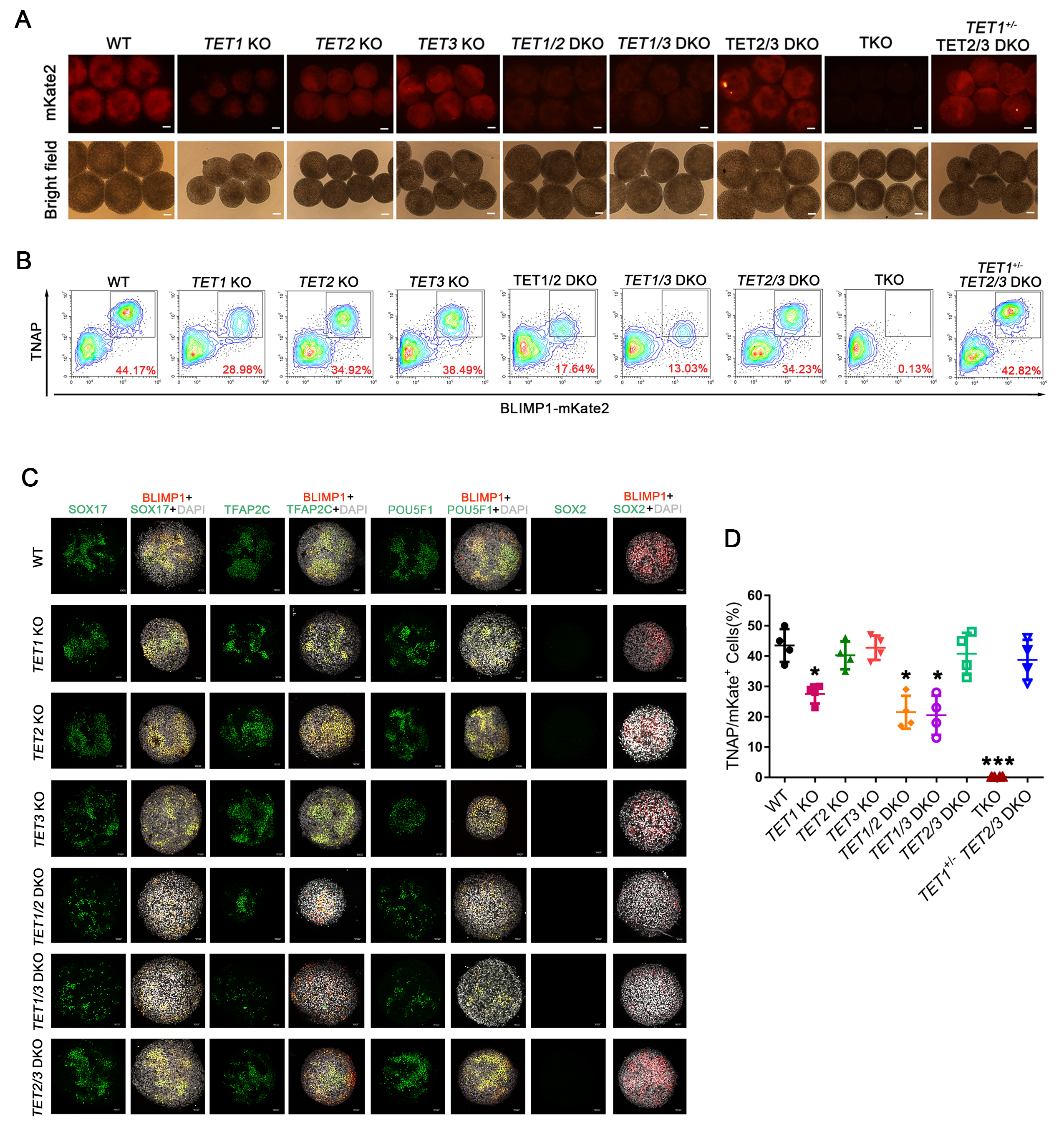

Supplement: Supplementary file 3 — Additional file 3: Fig. S3. hPGCLCs Differentiation is Sensitive to TET Gene Dosage, Related to Fig. 1. (A) Bright field and fluorescence images of day 4 embryoids with BLIMP1-mKste2 reporter in each cell line, Scale bar = 200 μm; (B) Representative FACS plots of TNAP/BLIMP1 positive cells at day 4 of hPGCLC differentiation for TET-knockout mutants; (C) Immunofluorescence of SOX17, TFAP2C, POU5F1, BLIMP1 and SOX2 at the day4 embryoids for TET-knockout mutants. Scale bar = 50 μm; (D) Quantification of FACS at day 4 of hPGCLC induction in TET-knockout mutants; n = 4 independent experiments. Data are presented as means ± s.d. Statistical analysis was performed by Student’s t-test (two-sided), compared to WT group *p < 0.05, ***p < 0.001. [file 13578_2022_917_MOESM3_ESM.tif]

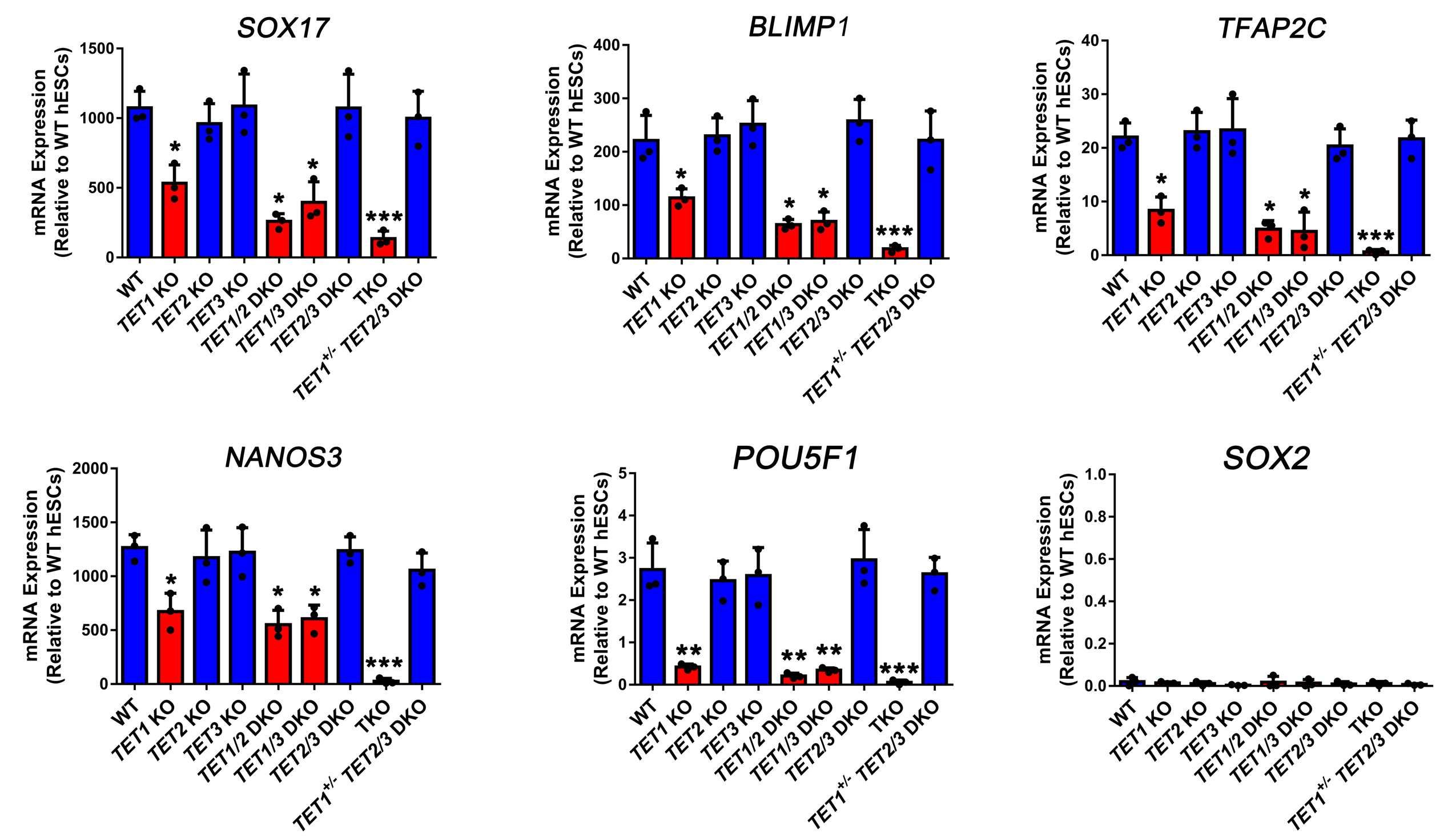

Supplement: Supplementary file 4 — Additional file 4: Fig. S4. RT-qPCR analysis for SOX17, BLIMP1, TFAP2C, NANOS3, POU5F1, SOX2 during hPGC differentiation in day 4 embryoids, Related to Fig. 1; n = 3 independent experiments. Data are presented as means ± s.d. Statistical analysis was performed by Student’s t-test (two-sided), compared to WT group, *p < 0.05, **p < 0.01, ***p < 0.001, Related to Fig. 1. [file 13578_2022_917_MOESM4_ESM.tif]

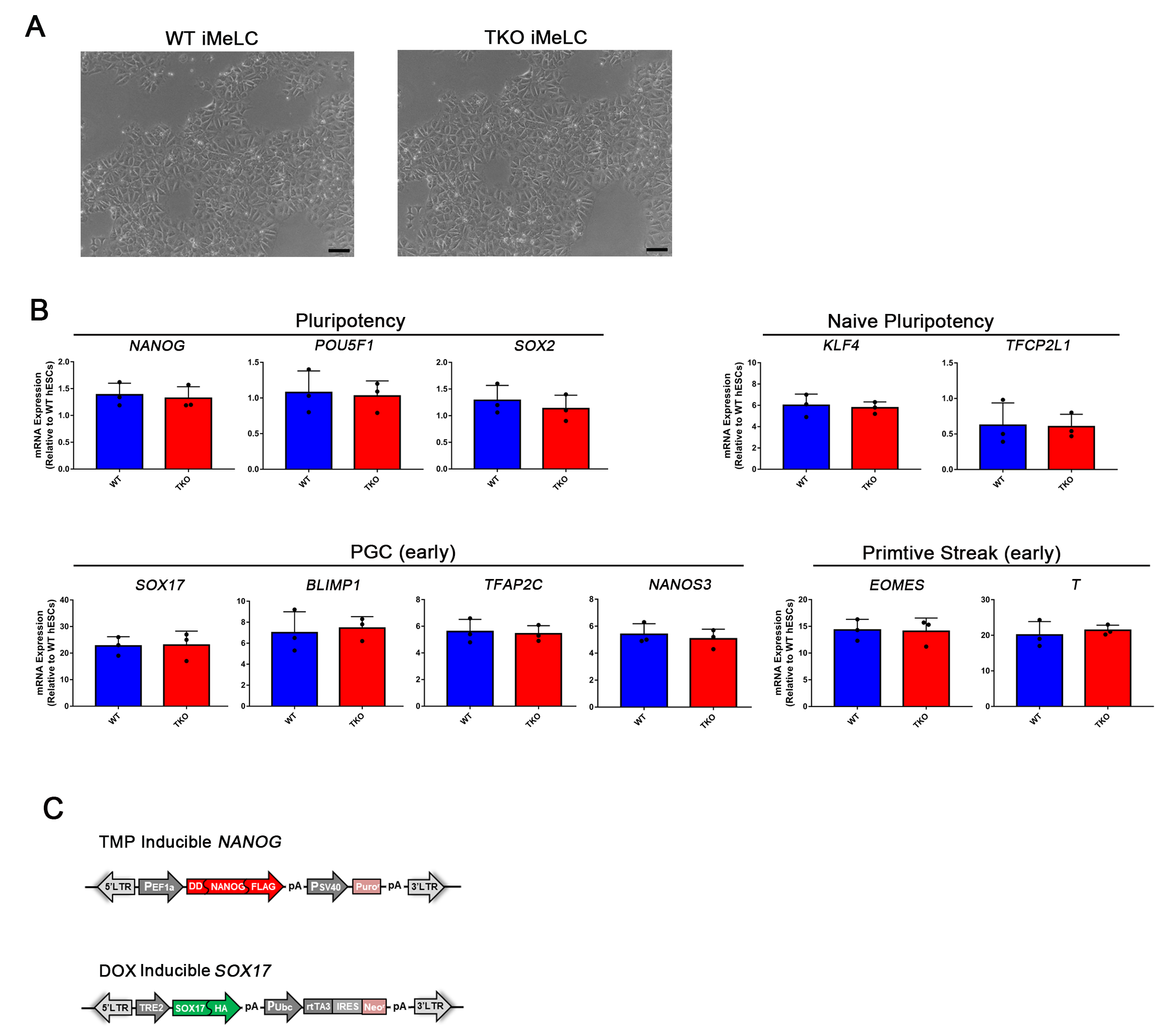

Supplement: Supplementary file 5 — Additional file 5: Fig. S5. TET Gene Knockout Shows No Effect on iMeLCs Induction, Related to Fig. 1. (A) Bright filed of iMeLCs induction from WT and TKO hESCs (42 h); (B) RT-qPCR analysis for each gene during iMeLCs differentiation in 42 h; n = 3 independent experiments. Data are presented as means ± s.d; (C) Vectors for overexpression of DOX-inducible SOX17, and TMP-inducible NANOG transgenes in BLIMP1–mKate2 reporter hESCs. [file 13578_2022_917_MOESM5_ESM.tif]

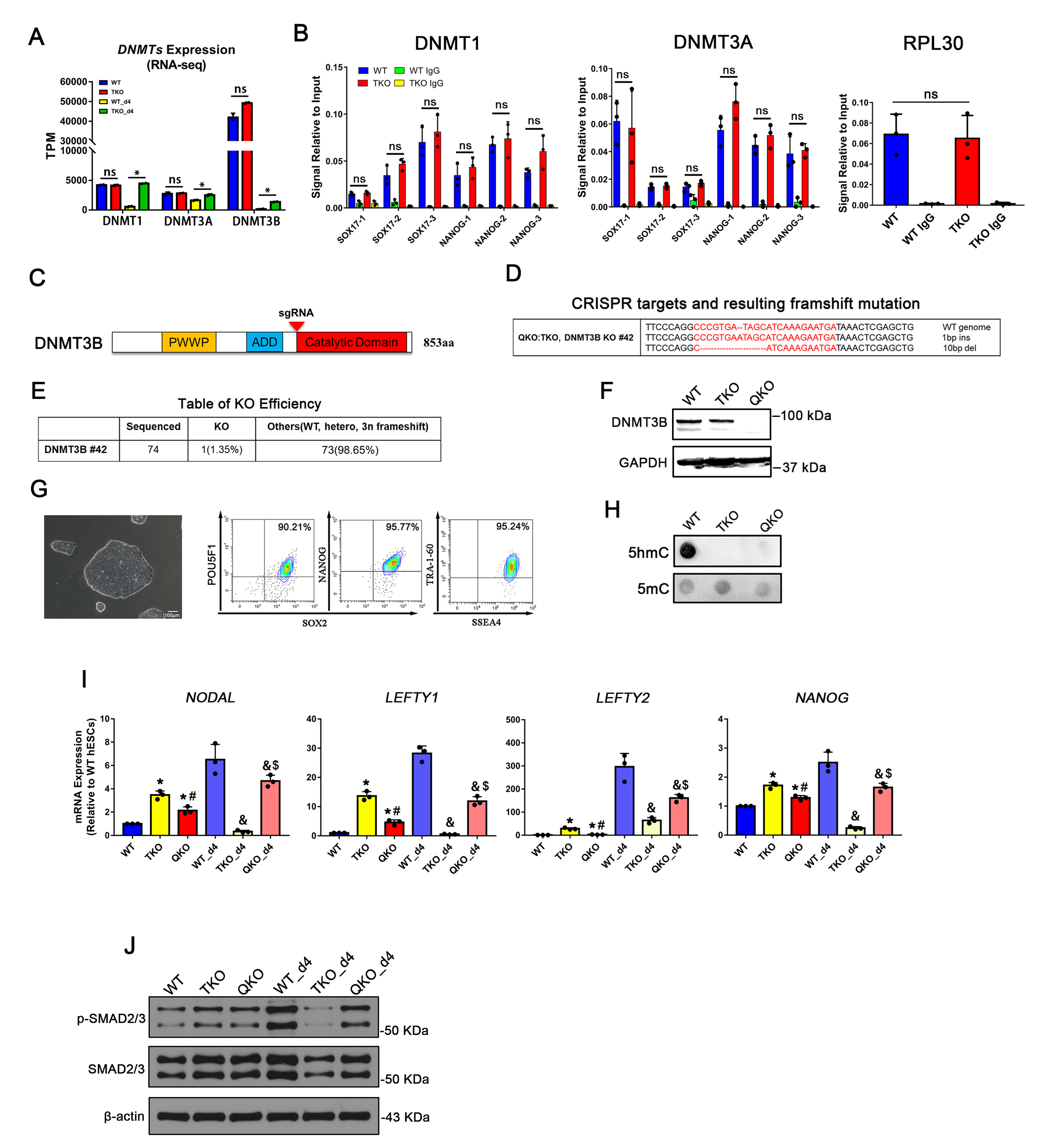

Supplement: Supplementary file 6 — Additional file 6: Fig. S6. DNMT3B Deletion Partially Rescues Hypermethylation of NANOG and SOX17 Promoters in TKO hESCs, Related to Fig. 5. (A) RNA-seq analysis of DNMT1, DNMT3A and DNMT3B in WT hESCs, TKO hESCs, WT d4 PGCLCs and TKO day4 embryoids; n = 2 independent experiments. Data are presented as means ± s.d. Statistical analysis was performed by Student’s t-test (two-sided); (B) ChIP–qPCR for DNMT1 and DNMT3A at the SOX17 and NANOG promoters in WT and TKO hESCs, RPL30 was used as positive control supplied in ChIP kit; n = 3 independent experiments. Data are presented as means ± s.d. Statistical analysis was performed by Student’s t-test (two-sided); (C) Design of the CRISPR targets for DNMT3B genes, using gRNAs (red arrows) that target the sequences corresponding to the beginning of the catalytic domain in DNMT3B; (D) The DNA sequences of both alleles for the indicated knockout lines. Red letters indicate the positions of the guide RNAs. del: deletion; ins: insertion; (E) The efficiency for the homozygous knockouts of the TET alleles. The knockouts were confirmed as bi-allelic frame-shift nonsense mutations. The others include wild-types or heterozygous mutants, or alleles with deletions/insertions of 3 × N base pairs; (F) Western blots showing DNMT3B expression level in WT, TKO, QKO hESCs; (G) Left, A phase-contrast image of QKO hESCs. Scale bar = 100 μm. Right, FACS analysis for POU5F1, SOX2, NANOG, TRA-1–60, and SSEA-4 expression in QKO hESCs; (H) Analysis of 5hmC and 5mC levels in WT, TKO, QKO hESCs by dot blot; (I) mRNA expression was assayed in day4 embryoids using qRT-PCR assay; n = 3 independent experiments. Data are presented as means ± s.d.; Statistical analysis was performed by Student’s t-test (two-sided), * represent compared to WT group p < 0.05, # represent compared to TKO group p < 0.05, & represent compared to WT_d4 group p < 0.05, $ represent compared to TKO_d4 group p < 0.05; (J) Western blots showing SMAD2/3 and p-SMAD2/3 expression level [file 13578_2022_917_MOESM6_ESM.tif]

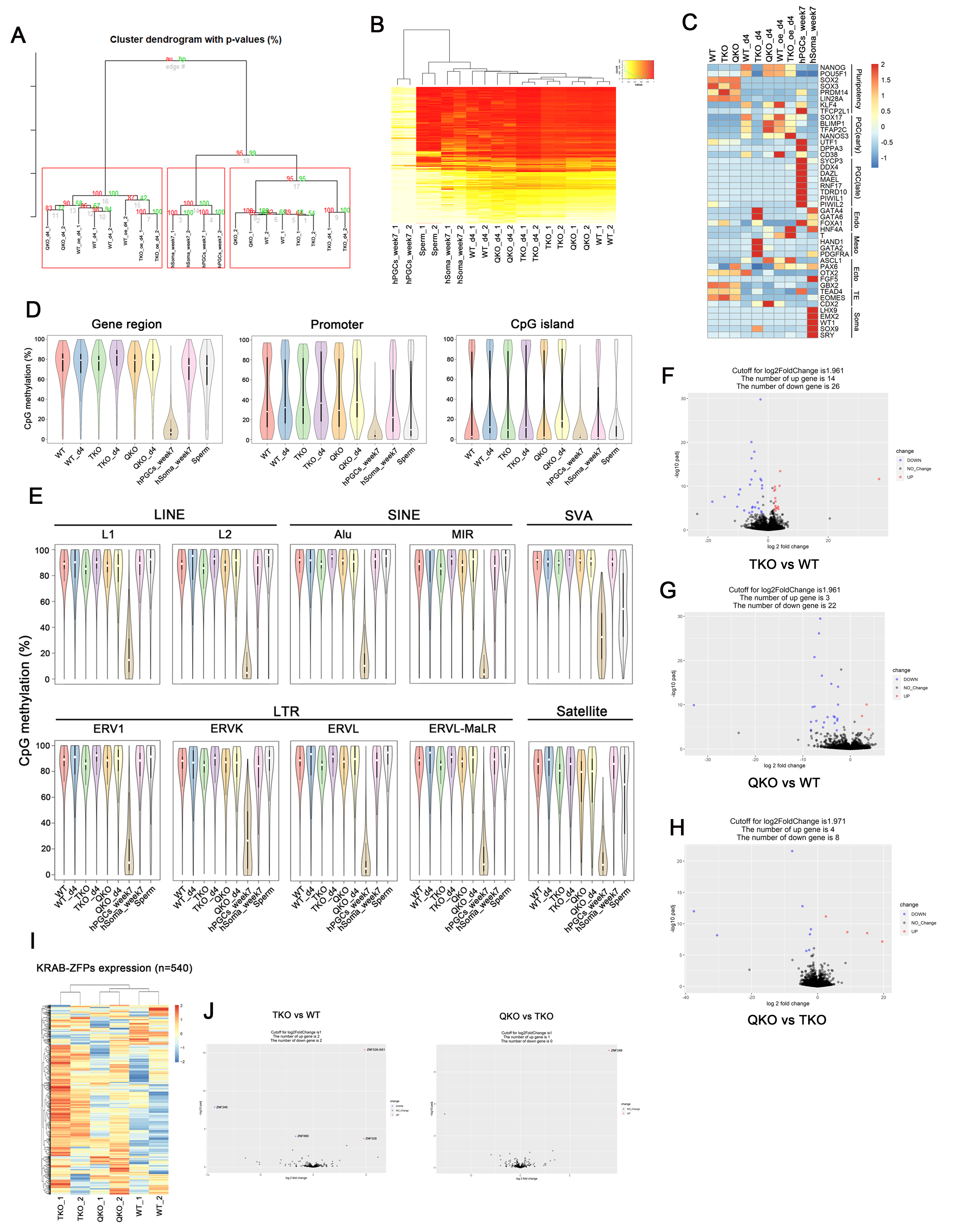

Supplement: Supplementary file 7 — Additional file 7: Fig. S7. Transcription and DNA methylation Profile of Each Cell Lines, Related to Fig. 6. (A) Unsupervised hierarchical clustering of the transcriptomes (two independent experiments) of each sample, AU (Approximately Unbiased) p-value and BP (Bootstrap Probability) value; (B) Hierarchical clustering on the top changed 1000 promoters by RnBeads; (C) Heat map of key PGC-associated genes (early and late), pluripotency, mesoderm, endoderm, and gonadal somatic (Soma) markers; (D) Violin plots showing distribution of CpG methylation in gene region, promoter and CpG island, white point indicates median; (E) Violin plots showing distribution of CpG methylation in major human repetitive elements classes and families, white point indicates median; (F) Volcano plot of RNA-seq data illustrating transcriptional changes in TKO as compared to WT hESCs; n = 2 independent experiments; (G) Volcano plot of RNA-seq data illustrating transcriptional changes in QKO as compared to WT hESCs; n = 2 independent experiments; (H) Volcano plot of RNA-seq data illustrating transcriptional changes in QKO as compared to TKO hESCs; n = 2 independent experiments; (I) Unsupervised hierarchical clustering of KRAB-ZFPs expressions in WT, TKO, QKO hESCs; (J) Volcano plot of KRAB-ZFPs expressions illustrating transcriptional changes in TKO as compared to WT hESCs, and QKO as compared to TKO hESCs; n = 2 independent experiments. [file 13578_2022_917_MOESM7_ESM.tif]

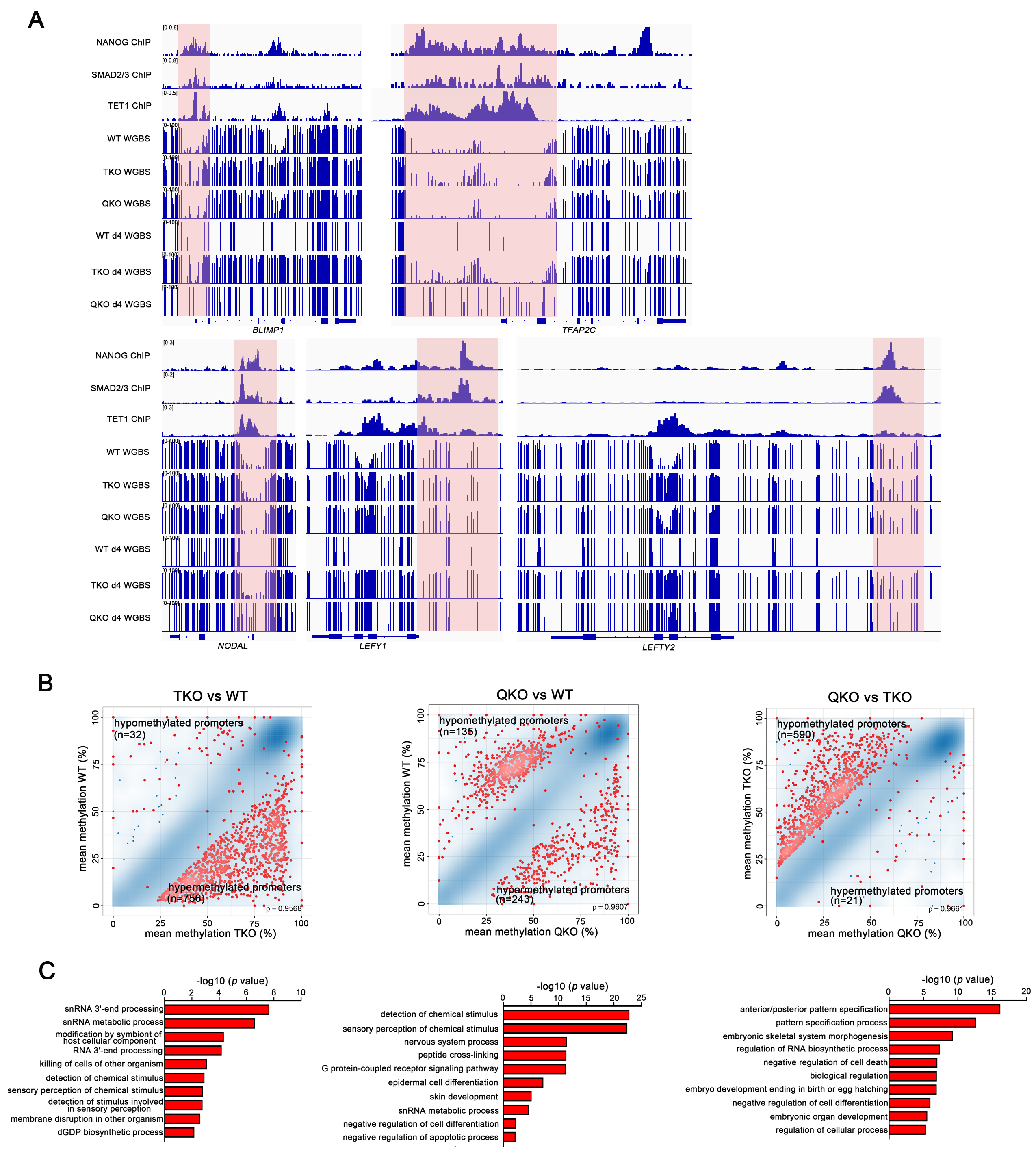

Supplement: Supplementary file 8 — Additional file 8: Fig. S8. Differential Methylation Promoters and GO Analysis, Related to Fig. 7. (A) NANOG, SMAD2/3, TET1 binding sites and methylation profile for the BLIMP1, TFAP2C, NODAL, LEFTY1 and LEFTY2 locus, the red area indicated promoter region; (B) Density-scatterplot showing differentially methylated promoters in each cell line; (C) GO analysis of hypomethylation promoters in Fig. S8B. [file 13578_2022_917_MOESM8_ESM.tif]
