# Supplementary material for: The balance between NANOG and SOX17 mediated by TET proteins regulates specification of human primordial germ cell fate
Source: Cell Biosci. 2022 Nov 4;12:181. doi: 10.1186/s13578-022-00917-0 (PMC9636699; doi:10.1186/s13578-022-00917-0)
Supplement: Supplementary file 9 — Additional file 9: Tables S1–S7. [file 13578_2022_917_MOESM9_ESM.docx]

Supplementary Tables

Supplementary Table 1. CRISPR sgRNA sequences used for inactivation of *TET1*, *TET2*, *TET3* and *DNMT3B* genes.

| Gene Targeted | sgRNA Sequence (5’-3’) |
| --- | --- |
| *TET1* | GGCCCATATTATACACACCT |
| *TET2* | CTTATGGTCAAATAACGACT |
| *TET3* | GATCGAGAAGGTCATCTACA |
| *DNMT3B* | TCATTCTTTGATGCTATCAC |

Supplementary Table 2. Sanger sequencing of gene knockout PCR primers

| **Name** | **Forward primer 5’-3’** | **Reverse primer5’-3’** |
| --- | --- | --- |
| *TET1* | GGGGCAGGAAGAAAGGAACA | TGCCAGACAAGCAATGGGAA |
| *TET2* | ACACAGCAACCCCAAACTGA | TCACAAGACACAAGCATCGGT |
| *TET3* | GCTGTGTCTCCAGAGCCTTT | TGACAGCATCCCCAGTTTCC |
| *DNMT3B* | TAGCCCATCCTTGGCTTTGG | GAGGGCCAGCTGAAACTCTT |

Supplementary Table 3. RT-qPCR primers

| **Name** | **Forward primer 5’-3’** | **Reverse primer5’-3’** |
| --- | --- | --- |
| *GAPDH* | CGCTTCGCTCTCTGCTCCTCCTGT | GGTGACCAGGCGCCCAATACGA |
| *BLIMP1* | CGGGGAGAATGTGGACTGGGTAGAG | CTGGAGTTACACTTGGGGGCAGC |
| *KLF4* | TACCAAGAGCTCATGCCACC | CGCGTAATCACAAGTGTGGG |
| *NANOS3* | CCCGAAACTCGGCAGGCAAGA | AAGGCTCAGACTTCCCGGCAC |
| *TFAP2C* | CGCTCATGTGACTCTCCTGACATCC | TGGGCCGCCAATAGCATGTTCT |
| *POU5F1* | GCTGGAGCAAAACCCGGAGG | TCGGCCTGTGTATATCCCAGGGTG |
| *SOX2* | ACACCAA TCCCA TCCACACT | CCTCCCCAGGTTTTCTCTGT |
| *SOX17* total | GAGCCAAGGGCGAGTCCCGTA | CCTTCCACGACTTGCCCAGCAT |
| *SOX17* endo | CCTGGGTTTTTGTTGTTGCT | CCCCAAACTGTTCAAGTGG |
| *NANOG* total | TGCTGAGATGCCTCACACGGA | TGACCGGGACCTTGTCTTCCTT |
| *NANOG* endo | AGAGGTCTCGTATTTGCTGCAT | AAACACTCGGTGAAATCAGGGT |
| *TFCP2L1* | AGCTCAAAGTTGTCCTACTGCC | TTCTAACCCAAGCACAGATCCC |
| *GATA4* | TCCCTCTTCCCTCCTCAAAT | TCAGCGTGTAAAGGCATCTG |
| *EOMES* | CAACATAAACGGACTCAATCCCA | ACCACCTCTACGAACACATTGT |
| *T* | ACCCAGTTCATAGCGGTGAC | CCATTGGGAGTACCCAGGTT |
| *PAX6* | GCGGGTGACAAAATAGTTGTCTT | GCCAGGATGTCAAATCTCTCCA |
| *CDX2* | TCACCATCCGGAGGAAAGCC | CTCTCCTTTGCTCTGCGGTT |
| *HAND1* | GGGTTAAACAGGTCTTTGGGC | CCCTATTAACGCCGCTCCAT |
| *FOXA1* | AAGGCATACGAACAGGCACTG | TACACACCTTGGTAGTACGCC |

Supplementary Table 4. ChIP-qPCR and Epimark primers

| **Name** | **Forward primer 5’-3’** | **Reverse primer5’-3’** |
| --- | --- | --- |
| *SOX17-1* | AGAATGGACGCTCGGTATGT | AGGGAGACTCGAAAAGCCGT |
| *SOX17-2* | CCTTGGGCAAGTACGTCGAT | CGGGGTTAGGGAGACTCGAA |
| *SOX17-3* | TTGGCCACATCTGTGCAGAAAA | GTCGCGGTCTGGTCTACAGC |
| *NANOG-1* | TATTTGTTGCTGGGTTTGTCTT | AATTCTCAGTTAATCCCGTCTAC |
| *NANOG-2* | TGCCTTGGCTTCATGCTATA | TAGGGTGATTTCTTGATTTGAGA |
| *NANOG-3*  *NODAL*  *LEFTY1*  *LEFTY2* | CATCCTTAGTCCAGCCTGTTCC  TCCAGCTTGGGTTTGGTTGT  GGTAGGGATCACTCATTCCCTTG  GAGGGCTTTCAAATTTGGCTGG | GCTGGAAGGCCGACTTACTAC  AGCAATCACATGCACCCAGA  AATGGTCTGAAGTGTTGCCAGG  TCACAGCCTAGTGGTGTTCAGG |

Supplementary Table 5. Antibodies used in this study

| **Antibody** | **Company** | **Cat NO.** |
| --- | --- | --- |
| POU5F1 | BD Biosciences | 560589 |
| NANOG | BD Biosciences | 560589 |
| SOX2 | BD Biosciences | 560589 |
| TRA-1-60 | BD Biosciences | 560884 |
| SSEA4 | BD Biosciences | 560308 |
| TNAP | BD Biosciences | 561495 |
| 5 hmC | abcam | ab106918 |
| 5 mC | abcam | ab10805 |
| SOX17 | R&D Systems | AF1924 |
| BLIMP1 | R&D Systems | MAB36081 |
| TFAP2C | SantaCruz Biotechnology | sc-8977 |
| POU5F1 | abcam | ab181557 |
| SOX2 | abcam | ab92494 |
| DNMT3B | abcam | ab2851 |
| SMAD2/3 | Cell Signaling | #8685 |
| p-SMAD2/3 | Cell Signaling | #8828 |

Supplementary Table 6. Plasmid used in this study

| Names | Addgene Cat NO. |
| --- | --- |
| pX330-hSpCas9 | #42230 |
| pENTR1A no ccDB | #17398 |
| pInducer20 | #44012 |
| pLEX_307 | #41392 |
| pBMN DHFR-YFP | #29325 |

Supplementary Table 7. List of sequencing datasets analysised in this study

| Sample Name | Sample Type | GEO Dataset |
| --- | --- | --- |
| hPGC_week7 | RNA-seq | GSE60138 |
| hSoma_week7 | RNA-seq | GSE60138 |
| hPGC_week7 | BS-seq | SRP057098 |
| hSoma_week7 | BS-seq | SRP057098 |
| Sperm | BS-seq | GSE2986305 |
| Tet_TKO_E6.5_Epiblast | RNA-seq | GSE76261 |
| WT_E6.5_Epiblast | RNA-seq | GSE76261 |
| TET1 ChIP-Seq | ChIP-seq | GSE150072 |
| NANOG ChIP-seq | ChIP-seq | GSE109524 |
| SMAD2/3 ChIP-seq | ChIP-seq | GSE109524 |

Supplementary Table 8. RNA-seq result for all samples
